# Supplementary material for: Beyond body maps: Information content of specific body parts is distributed across the somatosensory homunculus
Source: Cell Rep. 2022 Mar 15;38(11):110523. doi: 10.1016/j.celrep.2022.110523 (PMC8938902; doi:10.1016/j.celrep.2022.110523)
Supplement: Document S1. Figures S1–S5 [file mmc1.pdf]

Cell Reports, Volume *38*

Supplemental information

Beyond body maps: Information content of specific body parts is distributed across the somatosensory homunculus

Dollyane Muret, Victoria Root, Paulina Kieliba, Danielle Clode, and Tamar R. Makin

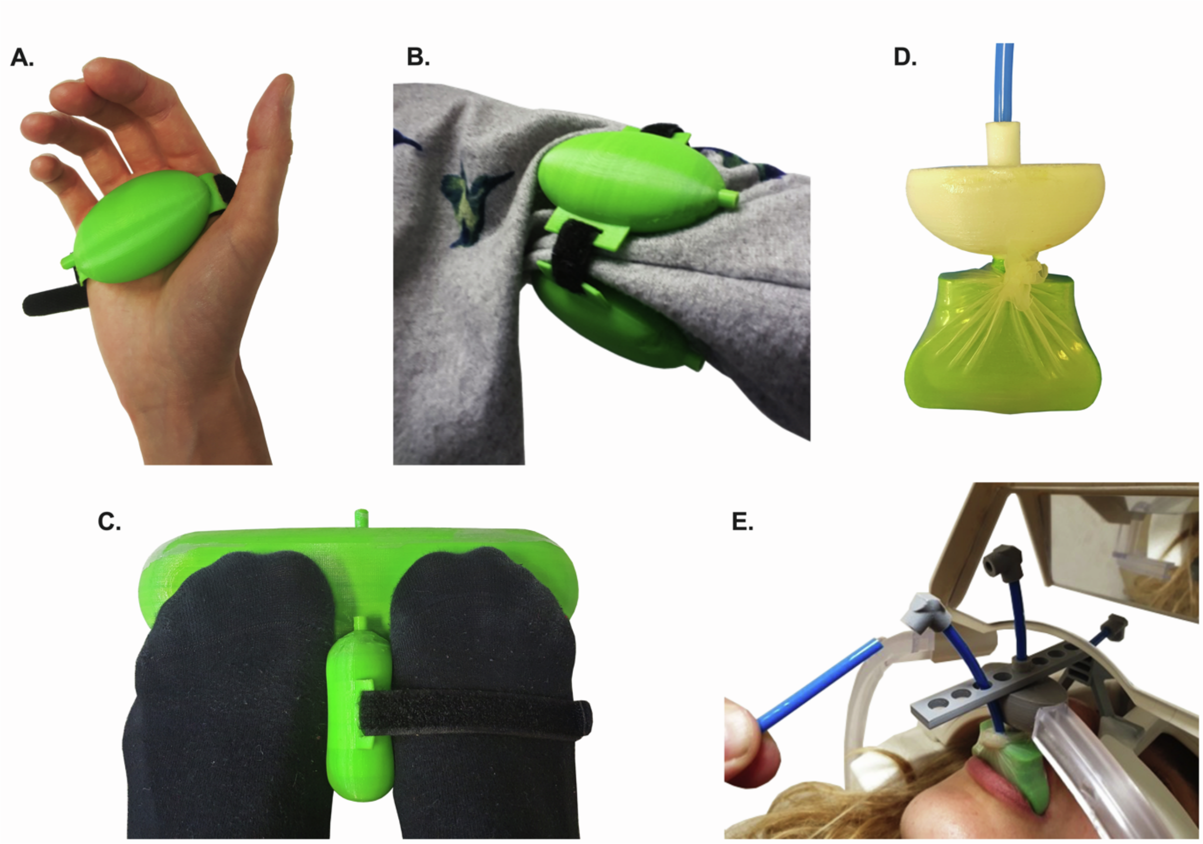

Figure S1. Objects used in the body task. Related to Figure 1, 4 and 5, and to STAR Methods. Silicon 3D printed objects (NinjaFlex© 3D printing filament) were placed and secured with straps in the participants dominant hand (A), around the non-dominant arm (B), between the feet (secured at the level of their right metatarsophalangeal joints), and behind their toes (strapped to a footrest secured on the bed behind their feet) (C). Participants were instructed to either squeeze (i.e., closing the fingers for the hand, against the torso for the arm and between the feet) or push (i.e., against the bed for hand and arm, and against the footrest for the feet) the object with each body part. Three participants also had an object placed in between their lips (D), and secured on a fully adjustable frame, fixed on the coil (E), object that could be either squeezed or pushed. The other participants were asked to either purse their lips (equivalent to squeezing) or blow kisses (equivalent to pushing). A plastic tube was connecting each of the objects to a transducer system in the control room, allowing us to monitor and quantify the amount of pressure exerted.

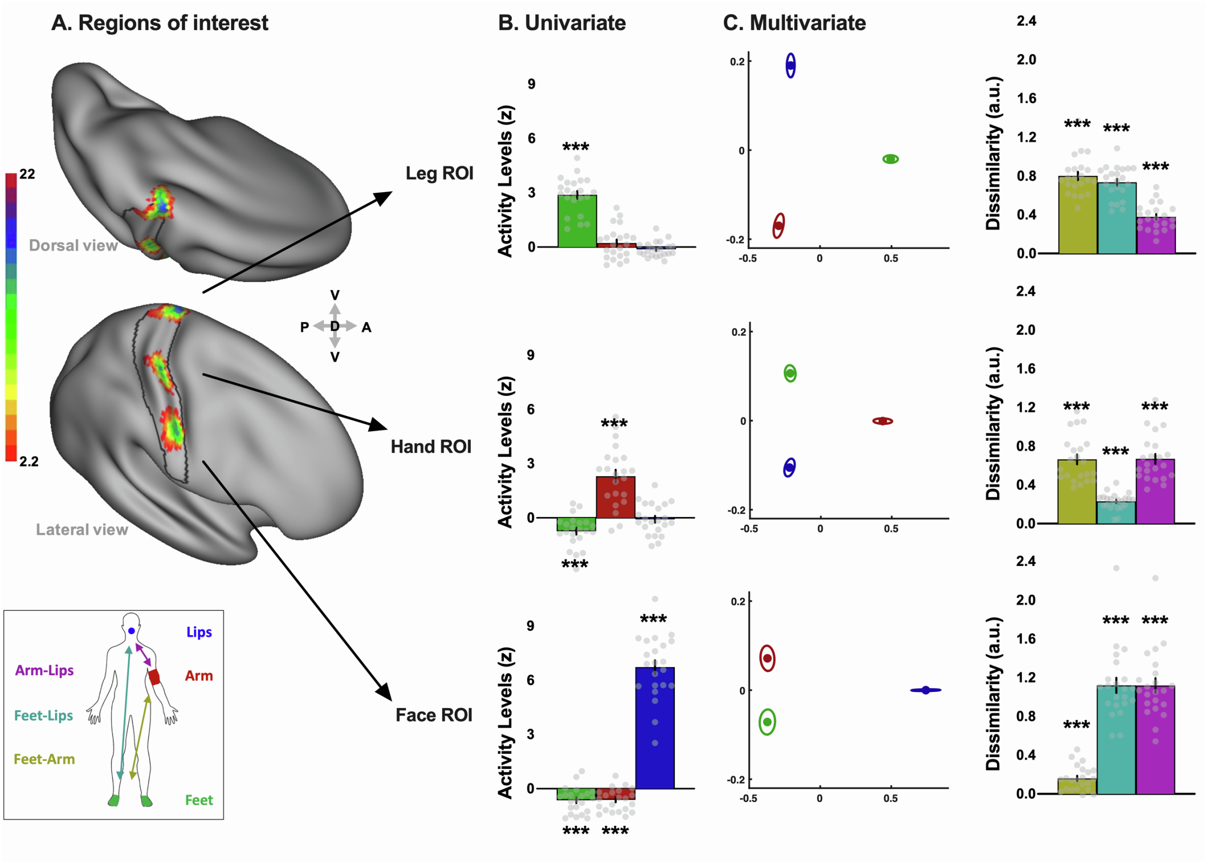

Figure S2. Regions of interest, selectivity, and multivariate information content related to specific body parts across S1 Homunculus in the non-dominant hemisphere. Related to Figure 1. All figure annotations are as denoted in Figure 1. Asterisks indicate a significant difference relative to zero; ***$p < 0.001$. In agreement with what was found in the dominant hemisphere (Fig. 1), ROIs in the non-dominant hemisphere were highly selective to their primary body parts, showing activity levels significantly above zero for this body part only (primary body parts: all $t \geq 6.50$, all $p < 0.001$, all $d \geq 1.39$ CI [0.88 1.87]; non-primary body parts: all $t \leq 1.20$, all $d \leq 0.25$ CI [-0.10 0.61]). And here again, the dissimilarity between activity patterns evoked by each movement were significantly greater than zero not only for pairs of body parts involving the primary body part of each ROI (all $t \geq 13.01$, all $p < 0.001$, all $d \geq 2.77$ 95% CI [1.97 ∞]), but also for pairs of body parts non-primary to the ROI (all $t \geq 5.84$, all $p < 0.001$, all $d \geq 1.25$ 95% CI [0.76 ∞]).

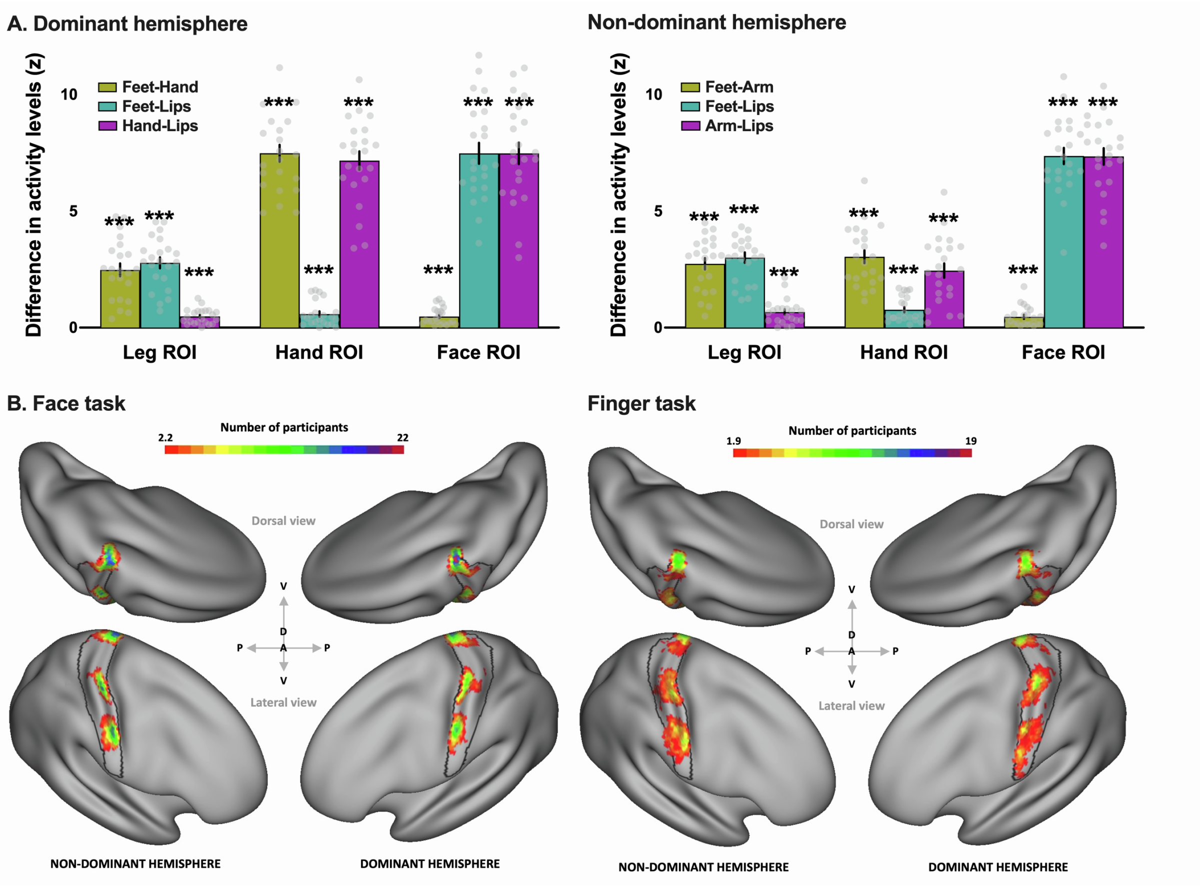

Figure S3. Univariate information content related to specific body parts across the S1 Homunculus and regions of interest for the face and finger tasks. Related to Figure 1A&C. A) Absolute differences between the univariate activity levels observed for each pair of body parts (yellow: feet-hand/arm, cyan: feet-lips, magenta: hand/arm-lips) across S1 ROIs in the hemisphere contralateral to the dominant hand (left) or non-dominant arm (right). In each hemisphere absolute differences were significantly greater than zero not only for pairs of body parts involving the primary body part of each ROI (all $t_{(21)} \geq 7.96$, all $p < 0.001$, all $d \geq 1.70$ 95% CI [1.13 ∞]), but also for pairs of body parts that were non-primary to the ROI (all $t_{(21)} \geq 5.18$, all $p < 0.001$, all $d \geq 1.10$ 95% CI [0.65 ∞]). Grey dots represent individual participants. ***$p < 0.001$. **B)** Consistency maps across participants of regions of interest within the primary somatosensory cortex (S1) used for the face (left) and finger (right) tasks respectively. Same legend as Fig. 1A and Fig. S2A.

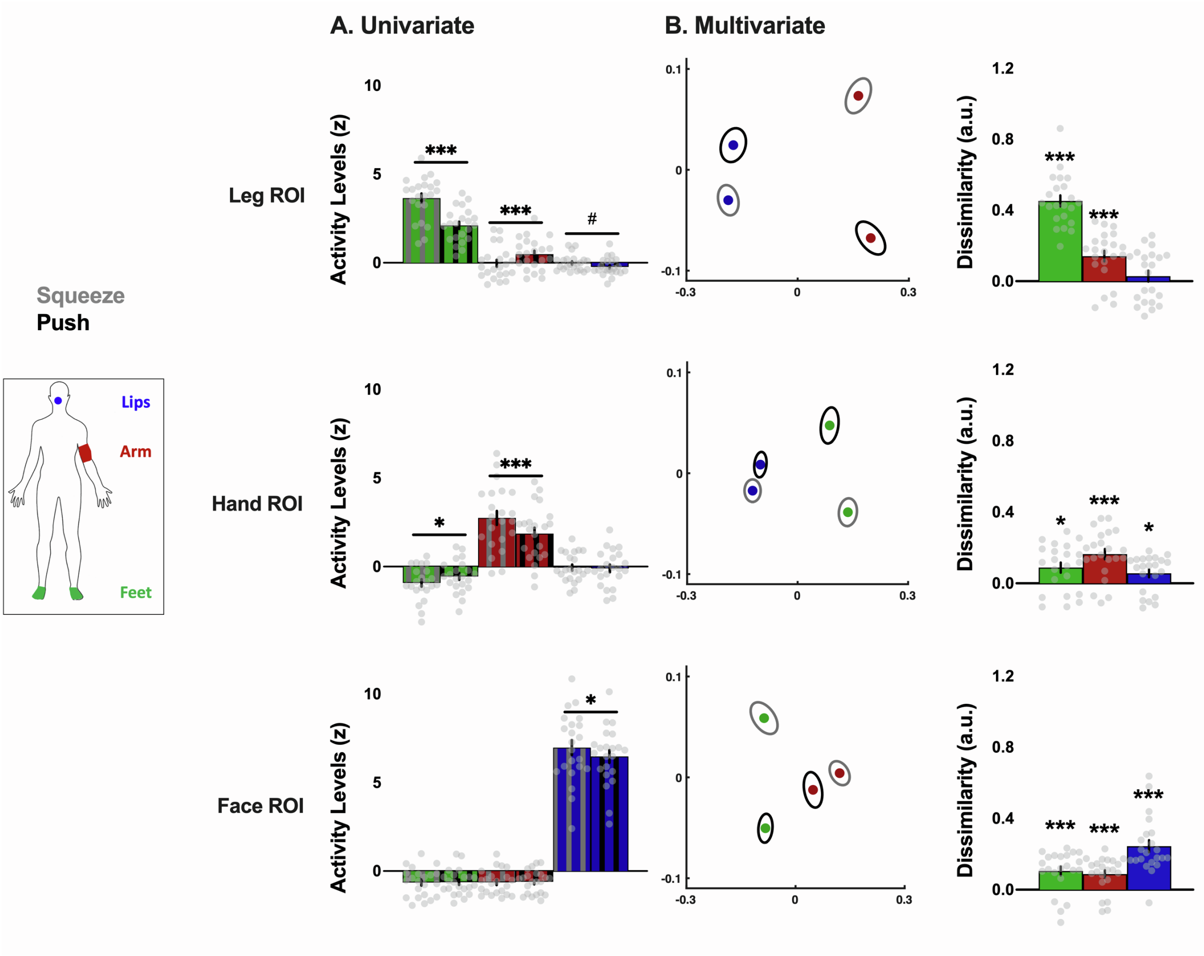

Figure S4. Univariate and multivariate content related to different actions performed with a given body part across S1 Homunculus in the non-dominant hemisphere. Related to Figure 4. All annotations are as denoted for Figure 4. *$p < 0.017$; #$p < 0.033$; ***$p < 0.001$. In agreement with what was found in the dominant hemisphere (Fig. 4), activity levels evoked by different actions were significantly different in the primary ROIs of each body part (all $p \leq 0.013$). Significant differences were also observed for arm movements in the Leg ROI ($t_{(21)} = -6.04$, $p < 0.001$) and for feet movements in the Hand ROI ($t_{(21)} = -2.67$, $p = 0.014$), and a trend was found for lip movements in the Leg ROI ($t_{(21)} = 2.44$, $p = 0.024$; other conditions: $-0.27 <$ all $t_{(21)} < 0.36$, all $p \geq 0.722$). Similar to the results observed in the dominant hemisphere, multivariate representational dissimilarities were significantly greater than zero not only in primary ROIs (all p values < 0.001, all $d \geq 0.99$ CI $[0.98\ \infty]$), but also in non-primary ROIs for arm and feet movements (all $p \leq 0.003$, all $d \geq 0.66$ CI $[0.26\ \infty]$), and for lip movements in the Hand ROI ($z_{(21)} = 196$, $p = 0.011$, $d = 0.55$ CI $[0.21\ \infty]$).

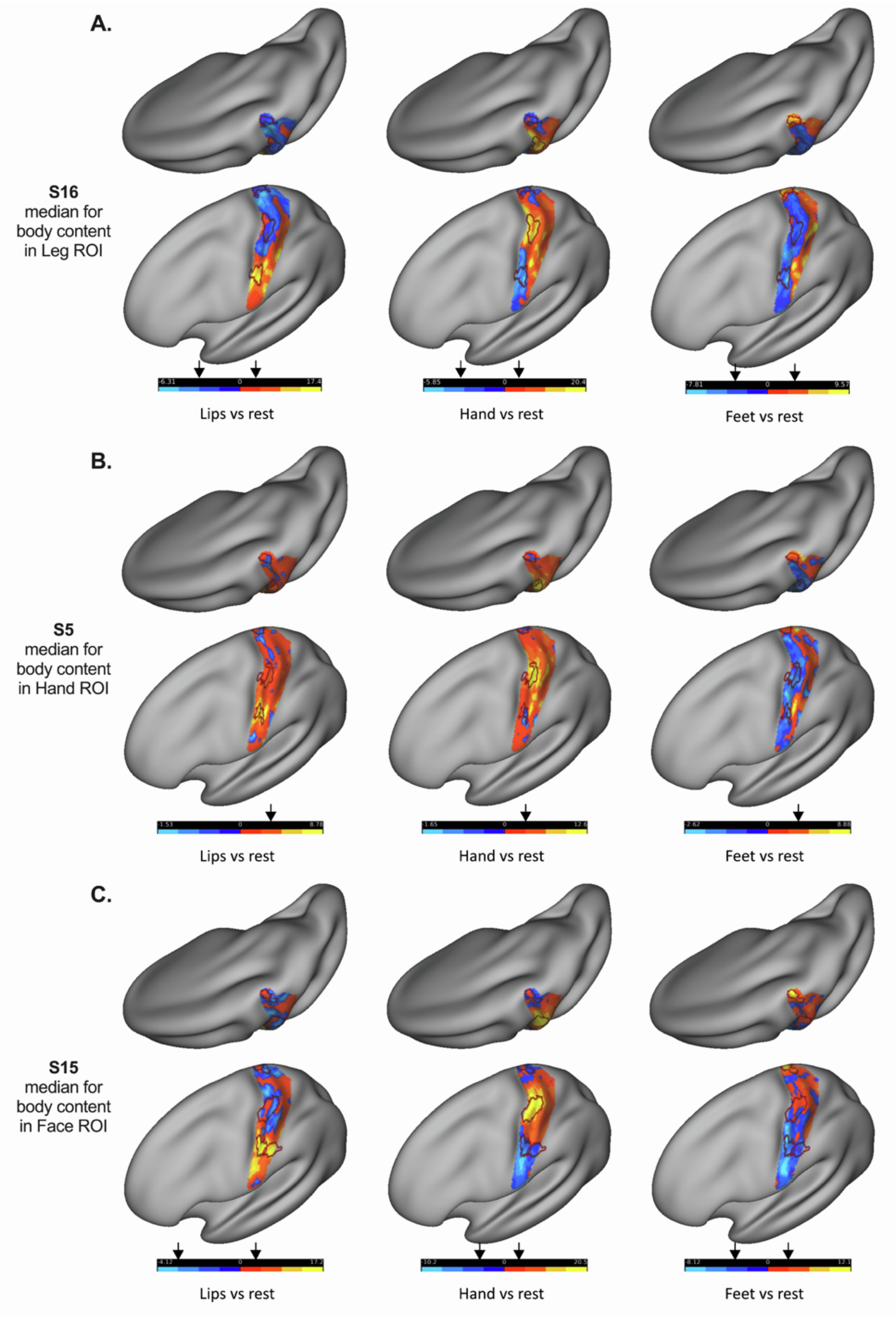

Figure S5. Example individual univariate activity maps obtained for different body parts across S1 in the dominant hemisphere. Related to Figure 1. Participants showing the closest dissimilarity to the group median for the body content in **A)** the Leg ROI, **B)** the Hand ROI and **C)** the Face ROI. Body content is defined as the dissimilarity between non-primary body parts. The colour scale codes for the z statistic (contrast vs rest), with positive bold responses represented in red-yellow and negative bold responses in blue. Standard activity threshold ($z = 3.1$) is indicated with the black arrows on the activity scales. Individual's ROIs are highlighted with red contours. The data is not thresholded and masked with S1 anatomical ROI. We note that the univariate activity profile was providing inconsistent results (across participants and paradigms), and thus these unthresholded maps of single participants should be interpreted with much caution.
