# Supplementary material for: Beyond body maps: Information content of specific body parts is distributed across the somatosensory homunculus
Source: Cell Rep. 2022 Mar 15;38(11):110523. doi: 10.1016/j.celrep.2022.110523 (PMC8938902; doi:10.1016/j.celrep.2022.110523)
Supplement: Document S2. Article plus supplemental information [file mmc2.pdf]

Cell Reports

Article

Beyond body maps: Information content of specific body parts is distributed across the somatosensory homunculus

Graphical abstract

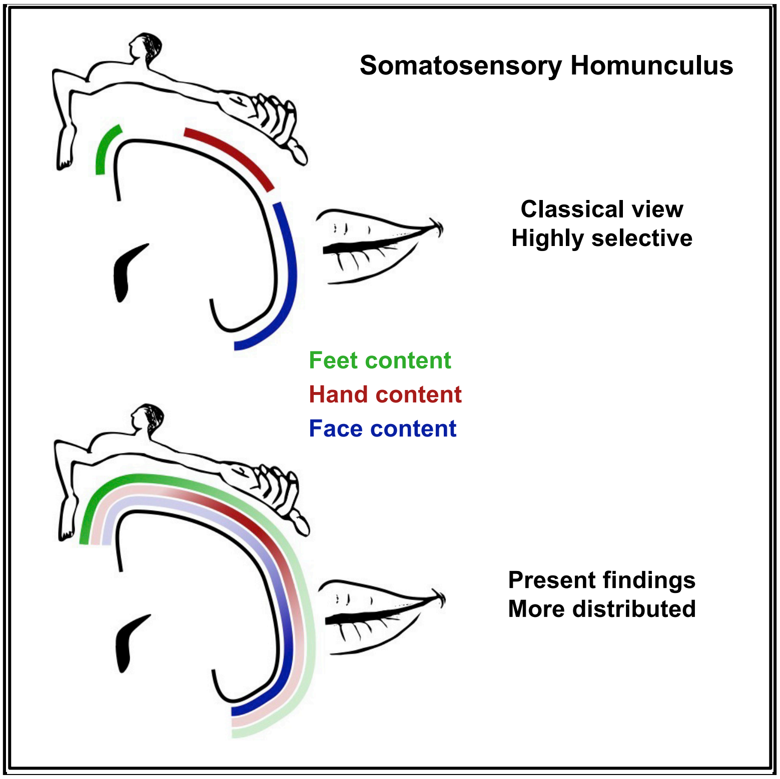

Highlights

- We replicate the high univariate selectivity profile of the somatosensory homunculus
- We use multivariate fMRI analysis to identify information content beyond selectivity
- Significant body part and action-related content are found throughout the homunculus
- Functional information is available, even in regions selective to other body parts

Authors

Dollyane Muret, Victoria Root, Paulina Kieliba, Danielle Clode, Tamar R. Makin

Correspondence

d.muret@ucl.ac.uk

In brief

The human body is represented in a topographic manner in the primary somatosensory cortex. Muret et al. show that the information related to each body part is not limited to its highly selective region within the topographic map and is instead more broadly distributed than previously considered.

Muret et al., 2022, Cell Reports *38*, 110523
March 15, 2022 © 2022 The Authors.
https://doi.org/10.1016/j.celrep.2022.110523

CellPress

Cell Reports

CellPress
OPEN ACCESS

Article

Beyond body maps: Information content of specific body parts is distributed across the somatosensory homunculus

Dollyane Muret,[1,5,*] Victoria Root,[1,2] Paulina Kieliba,[1] Danielle Clode,[1,3] and Tamar R. Makin[1,4]
[1]Institute of Cognitive Neuroscience, University College London, 17 Queen Square, London WC1N 3AZ, UK
[2]Wellcome Centre of Integrative Neuroimaging, University of Oxford, Oxford OX3 9DU, UK
[3]Dani Clode Design, 40 Hillside Road, London SW2 3HW, UK
[4]Wellcome Trust Centre for Neuroimaging, University College London, London WC1N 3AR, UK
[5]Lead contact
*Correspondence: d.muret@ucl.ac.uk
https://doi.org/10.1016/j.celrep.2022.110523

SUMMARY

The homunculus in primary somatosensory cortex (S1) is famous for its body part selectivity, but this dominant feature may eclipse other representational features, e.g., information content, also relevant for S1 organization. Using multivariate fMRI analysis, we ask whether body part information content can be identified in S1 beyond its primary region. Throughout S1, we identify significant representational dissimilarities between body parts but also subparts in distant non-primary regions (e.g., between the hand and the lips in the foot region and between different face parts in the foot region). Two movements performed by one body part (e.g., the hand) could also be dissociated well beyond its primary region (e.g., in the foot and face regions), even within Brodmann area 3b. Our results demonstrate that information content is more distributed across S1 than selectivity maps suggest. This finding reveals underlying information contents in S1 that could be harnessed for rehabilitation and brain-machine interfaces.

INTRODUCTION

Contrary to its motor counterpart, the primary somatosensory cortex (hereafter S1) is considered highly topographically organized, with relatively high levels of selectivity within each body part's representation (Schieber, 2001; Cunningham et al., 2013; Huber et al., 2020). This perspective over S1 organization arises from a long-lasting mapping tradition, initiated in the 19th century (Fritsch and Hitzig, 1870; Ferrier, 1873; Penfield and Boldrey, 1937) and continued since then in electrophysiology (Merzenich et al., 1978; Kaas et al., 1979; Baldwin et al., 2017), cortical stimulation (Roux et al., 2018; Sun et al., 2021), and neuroimaging studies (Nakamura et al., 1998; Germann et al., 2020; Saadon-Grosman et al., 2020; Willoughby et al., 2021). This conventional mapping approach assigns brain function to a given cortical area by selecting the most responsive body part for a set of neurons or voxels in a winner-takes-all manner. While this approach has been hugely beneficial, e.g., for understanding (Kaas et al., 1979) and restoring (Bensmaia and Miller, 2014; Flesher et al., 2016; Armenta Salas et al., 2018; Chandrasekaran et al., 2021) brain function, it may also eclipse the presence of other organizing principles that may also bear relevance for S1 function. In particular, the underlying (weaker) inputs that tend to be neglected in mapping approaches may also provide functional contributions, even if secondary to the dominant input.

While the organizing principles of S1 remain so far relatively unquestioned, other intrinsic organizing principles beyond somatotopy, e.g., representation of ethologically relevant actions, were suggested to underlie the general organization of body-related motor maps in the primary motor cortex (hereafter M1) (Graziano and Aflalo, 2007). Yet we know that M1 and S1 are tightly coupled, both anatomically and functionally (Matyas et al., 2010; Catani et al., 2012; Kumar et al., 2019). For instance, recent evidence in rodents (Auffret et al., 2018; Halley et al., 2020) and non-human primates (Widener and Cheney, 1997; Baldwin et al., 2018; Mayer et al., 2019) demonstrate that S1 stimulation can evoke movements or affect muscle activity. Active touch and habitual motor behavior were also suggested to impact S1 organization (Dempsey-Jones et al., 2019; Cybulska-Klosowicz et al., 2020). Moreover, the M1 and S1 hand regions were found to share similar representational features (Ejaz et al., 2015; Wesselink et al., 2019), which are in both cases better explained by inter-finger co-use in daily life than by topographic organization (Ejaz et al., 2015). Thus, despite their physiological differences, S1 and M1 may share more functional organization than previously thought, especially in terms of information content. This raises the question of whether S1 could also contain additional representational patterns underlying its topographic organization.

Recent evidence indeed points toward a more complex organization of S1, beyond its topographic organization. For example, an imaging study of the negative blood-oxygen-level-dependent

This is an open access article under the CC BY license (http://creativecommons.org/licenses/by/4.0/).

(BOLD) responses in humans revealed the presence of an underlying inverted homunculus (Tal et al., 2017), similar to M1 (Zeharia et al., 2012), suggesting a distribution of activity patterns across the homunculus. In addition, recent reports in rodents show that the information content arising from different tactile inputs provided to a digit could be decoded even from a non-adjacent digit representation (Enander and Jörntell, 2019). Other recent evidence in humans hints at the existence of distributed patterns of functional connectivity throughout S1 (Ngo et al., 2021; Thomas et al., 2021), as well as distributed processing underlying and interrelating finger representations. For example, focal anesthesia of a finger was found to affect the representation of all fingers (Wesselink et al., 2020) and intraneural microstimulation of single afferent units elicited widespread activity in the S1 hand region (Sanchez Panchuelo et al., 2016). Altogether, these recent reports stress the need to investigate the distribution of representational information content throughout S1 homunculus.

Recent methodological advancement (e.g., multivariate pattern analysis) allows to identify representational features beyond selectivity and thus provide an opportunity to reassess the homunculus. Here, we take advantage of these methods to investigate whether information content can be identified in S1 beyond the primary region of a given body part, as defined by conventional mapping criteria. We asked healthy participants to perform a series of sensorimotor paradigms in the scanner: (1) individual finger movements (hereafter finger task), (2) movements of specific facial parts (hereafter face task), or (3) two different actions (i.e., squeeze or push an object) with each of three body parts (i.e., lips, hand, and feet; hereafter body task). Using conventional univariate analyses on an independent dataset, we first defined individual S1 regions of interest showing high selectivity to face, hand, and foot movements. We then used representational similarity analysis (RSA) to index information content by quantifying multivoxel representational dissimilarities between actions and body parts. Cross-validated Mahalanobis distances provide a quantification of these dissimilarities, where distances that are greater than zero reflect significant information content (note that we deliberately avoid the term "representational content," since it could imply functional relevance). We found task-relevant information content was distributed across S1, demonstrating an intrinsic organization to S1 beyond somatotopy.

RESULTS

Our main approach was to identify distant and highly selective regions of the S1 homunculus at the individual participant level to assess their univariate activity levels and multivariate information content. Toward this end, within an S1 anatomical landmark (black contours in Figure 1A), we defined three regions of interest (ROIs) for each participant in each hemisphere, based on the 50 most selective voxels from an independent body localizer of the foot, hand, and face (see Figures 1A, S2A, and S3B for consistency maps across participants).

Information from different body parts is distributed across S1

We first focused on the body task to assess how information from different body parts is distributed across S1. To confirm the selectivity of our independent ROIs (Figure 1A), we extracted the average univariate activity level obtained for each body part (movement versus rest) in the contralateral hemisphere and found that each ROI was highly selective to its primary body part, showing significant activity for this body part only (one-sample t tests versus zero, primary body parts: all $t_{(21)} \geq 10.67$, all $p < 0.001$, all $d \geq 2.28$, 95% confidence interval [CI] [1.59, 2.93]; non-primary body parts: all $t_{(21)} \leq 1.00$, all $d \leq 0.21$, 95% CI [−0.14, 0.57]; Figure 1B). We then used RSA to quantify the dissimilarity between activity patterns evoked by each movement (see Figure S3A for similar analysis using the absolute difference between univariate activity levels). One-sample t tests with Bonferroni-corrected alpha levels ($\alpha = 0.017$, corrected for the three comparisons across body parts) confirmed that the representational dissimilarities were significantly greater than zero for pairs of body parts involving the primary body part of each ROI (all $t_{(21)} \geq 16.21$; all $p < 0.001$; all $d \geq 3.45$; 95% CI [2.50, ∞]). Interestingly, significant dissimilarities were also found for cortically remote pairs of body parts, where both body parts were non-primary to the ROI (in leg ROI: hand-lips: $t_{(21)} = 9.93$, $p < 0.001$, $d = 2.12$, 95% CI [1.46, ∞]; in hand ROI: feet-lips: $t_{(21)} = 6.51$, $p < 0.001$, $d = 1.39$, 95% CI [0.88, ∞]; in face ROI: feet-hand: $t_{(21)} = 10.59$, $p < 0.001$, $d = 2.26$, 95% CI [1.57, ∞]; Figure 1C). Thus, despite being highly selective to their primary body part, each ROI contained robust information content about non-primary body parts. This first evidence suggests that non-primary and cortically distant body parts may contribute information to a given region of the homunculus.

Information from different body subparts is distributed across S1

We next assessed how information from different body *subparts* is distributed across S1. Two tasks were used for that purpose: a face task involving bilateral movements and a finger task performed with each hand (see STAR Methods). First, to verify the selectivity of the individual ROIs used for each task, we quantified the univariate activity level obtained for each subpart (versus rest) in the contralateral ROIs. Alpha was adjusted to 0.013 and 0.01, corrected for four and five comparisons (respectively) across face and hand subparts. Activity levels (averaged across hemispheres; see STAR Methods) were significantly greater than zero for all face subparts in the face ROI (face ROI: all $t_{(21)} \geq 3.51$; all $p \leq 0.002$; all $d \geq 0.75$; 95% CI [0.34, 1.14]; Figure 2A, blue) and for the five fingers in the hand ROI (all $t_{(18)} \geq 7.44$; all $p < 0.001$; all $d \geq 1.71$; 95% CI [1.09, 2.29]; Figure 2A, red). Face subparts did not significantly activate the hand and leg ROIs (all $t_{(21)} \leq -0.86$; all $d \leq -0.18$; 95% CI [−0.54, 0.17]; Figure 2A, blue). For some fingers, significant positive activity levels (or trends) were found in the face and leg ROIs, as shown in red in Figure 2A (significant fingers: all $t_{(18)} \geq 2.70$, all $p \leq 0.015$, all $d \geq 0.62$, 95% CI [0.20, 1.02]; other fingers: all $t_{(18)} \leq 2.47$, all $p \geq 0.024$, all $d \leq 0.57$, 95% CI [0.15, 0.97]). Thus, while the face task shows high selectivity to the face ROI, finger-related activity seems to be more distributed across ROIs, though activity levels were still low on average, i.e., below the liberal 2.3 threshold usually used to threshold individual data (dotted line in Figure 2A).

CellPress
OPEN ACCESS

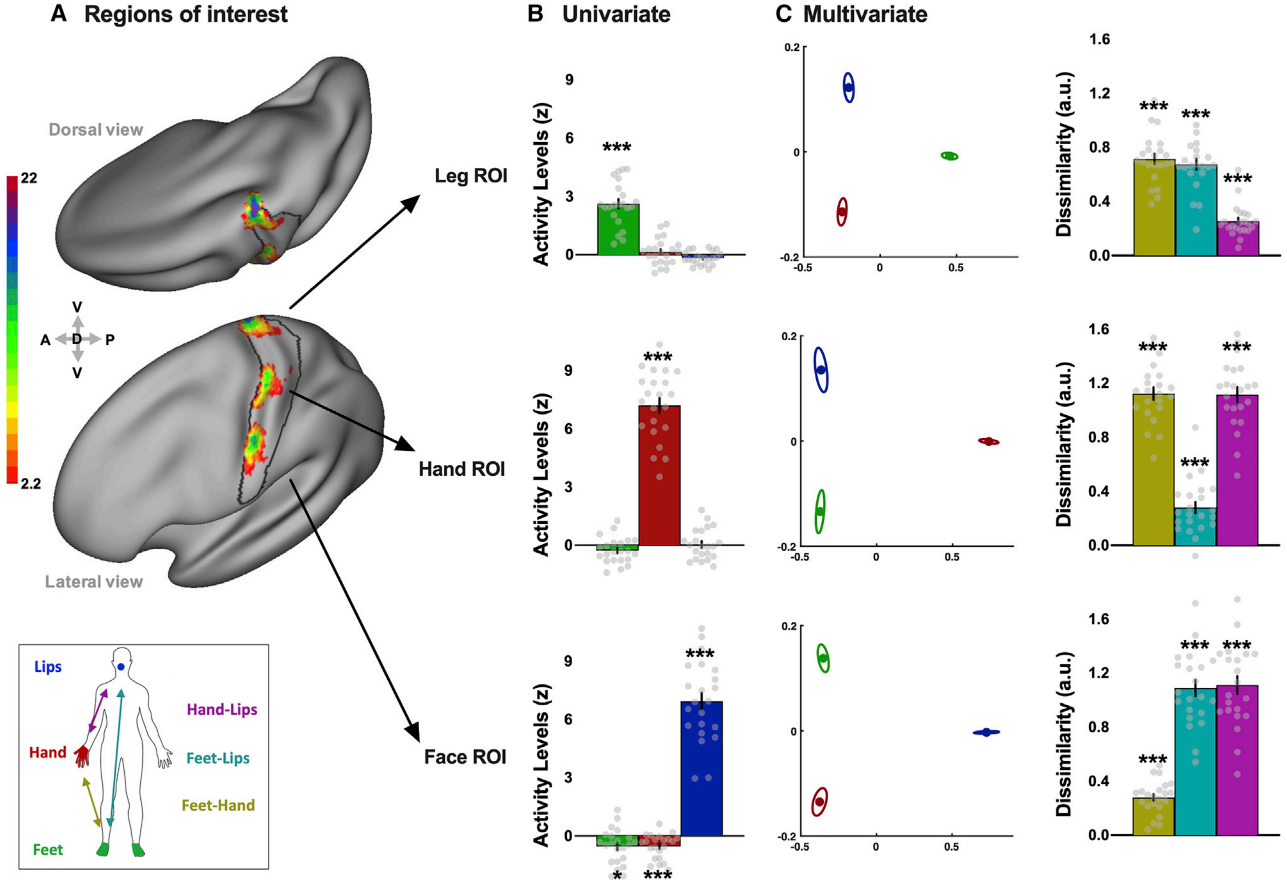

Figure 1. Regions of interest, selectivity, and multivariate information content related to specific body parts across S1 homunculus

(A) Consistency maps across participants of the S1 regions of interest (ROIs) for the body task (n = 22). Individual ROIs in the hemisphere contralateral to the dominant hand were converted to Montreal Neurological Institute (MNI) space and projected onto an inflated surface. The color code represents the number of participants with overlapping ROIs in the standard MNI space. The black contour shows the anatomical delineation of S1 used to restrict the ROI definition, based on a probabilistic atlas.

(B) Univariate activity levels (versus rest) for the three body parts (green, feet; red, hand; blue, lips) within each ROI. Only the primary body part of each ROI exhibited activity levels significantly above zero.

(C) Multivariate dissimilarities. The left plots are a multidimensional scaling (MDS) depiction of the representational dissimilarity between the three body parts (green, feet; red, hand; blue, lips) in each ROI. Ellipses indicate between-participant standard errors. The right histograms show the cross-validated dissimilarity (a.u.) observed for the three pairs of body parts in each ROI (yellow, feet-hand; cyan, feet-lips; magenta, hand-lips).

Data are represented as mean ± SEM. Gray dots represent individual participants. Asterisks indicate a significant difference relative to zero; *p < 0.017; ***p < 0.001. See Figure S2 for similar analysis in the non-dominant hemisphere and Figure S5 for example individual univariate activity maps.

We then investigated the multivariate pattern of dissimilarity between the four facial subparts (Figure 2B, blue) and the five fingers (Figure 2B, red) across S1 ROIs. A qualitative observation of the multidimensional scaling (MDS) plots (Figure 2B) suggests that the representational structure of the face and hand, whose canonical representations are seen in their respective primary ROIs, is preserved across ROIs. This was confirmed by quantitative assessment of dissimilarities. To reduce the number of comparisons, cross-validated representational dissimilarities from different pairs of subparts (i.e., face parts or fingers) were grouped according to the subpart's cortical neighborhood (i.e., adjacent versus non-adjacent; see inset in Figure 3). Alpha was adjusted to 0.025 to account for two comparisons for the adjacent and non-adjacent dissimilarities. We found that, for both tasks, dissimilarities between subparts' activity patterns were all significantly above zero, regardless of their neighborhood (Figure 3B). This was true not only in their respective primary ROI (all $t \geq 12.41$; all $p < 0.001$; all $d \geq 2.85$; 95% CI [1.96, ∞]) but also in remote parts of the homunculus, such as the hand ROI or the leg ROI for the face task (all $t_{(21)} \geq 5.85$; all $p < 0.001$; all $d \geq 1.29$; 95% CI [0.80, ∞]; Figure 3B, blue) and the face ROI or the leg ROI for the finger task (all $t_{(18)} \geq 3.18$; all $p \leq 0.003$; all $d \geq 0.73$; 95% CI [0.29, ∞]; Figure 3B, red). These results replicate the previous observation that information about body parts is not restricted to their primary S1 region.

Topographic features from different body subparts are distributed across S1

To assess whether the topographical content was preserved throughout S1, we next investigated the univariate and

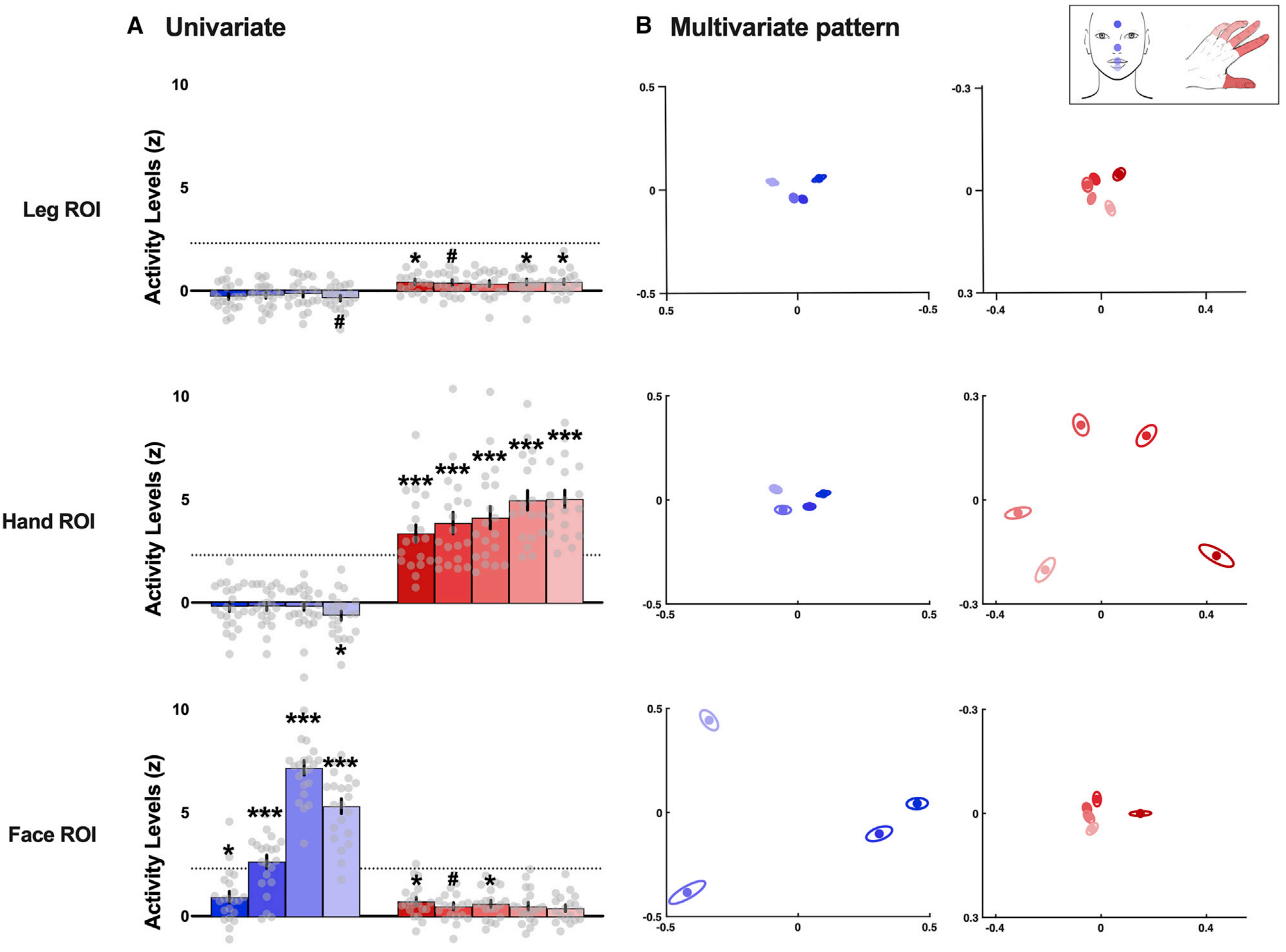

Figure 2. Selectivity and multivariate representational patterns of body subparts across S1 homunculus for face and finger tasks

(A) Univariate activity levels (versus rest) averaged across hemispheres obtained for the different subparts of the face task (shades of blue) and of the finger task (shades of red) within each ROI. The dotted line marks the 2.3 individual threshold.

(B) MDS plots illustrating the representational structure contained in the face (shades of blue) and hand (shades of red) activity across S1 ROIs (averaged across hemispheres). The canonical hand and face representational structures are observed, respectively, in the hand and face ROIs (i.e., primary ROIs).

Data are represented as mean ± SEM. Gray dots represent individual participants. *p < 0.013 and 0.010 for the face and finger tasks, respectively (alpha corrected for four and five comparisons, respectively); #p < 0.025 and 0.020 for the face and finger tasks, respectively; ***p < 0.001. The color code for the respective subparts is depicted in the inset.

multivariate differences between adjacent and non-adjacent subparts across ROIs for both the face and finger tasks. Univariate content was defined as the absolute difference between activity levels evoked by pairs of subparts in the different ROIs (see inset in Figure 3). A significant difference between adjacent and non-adjacent univariate content was found in the primary ROI of each task (both $t \geq -7.51$; both $p < 0.001$; both $d \geq -1.62$; 95% CI [−2.25, −0.97]; Figure 3A). In addition, a significant topographic difference was found for the face task in the hand ROI ($t_{(21)} = -3.30$; $p = 0.003$; $d = -0.70$; 95% CI [−1.16, −0.23]). No univariate topographic features were observed for other comparisons and ROIs (all $p \geq 0.099$; all $d \leq -0.40$; 95% CI [−0.86, 0.07]), despite the higher activity levels found for the finger than for the face task in non-primary ROIs ($t_{(39)} = -4.29$; $p < 0.001$; $d = -1.35$; 95% CI [−2.02, −0.66]; Figure 2A). Altogether, these results suggest that the univariate information content does not appear to be consistently topographically organized outside of its primary ROI.

We then compared the representational dissimilarities between adjacent and non-adjacent subparts, expecting adjacent subparts to be more similar if topographic information of body subparts is preserved across the homunculus. Similar to the univariate results, a significant difference between adjacent and non-adjacent subparts was found in the primary ROIs for both tasks (both $t \geq -13.59$; both $p < 0.001$; both $d \geq -2.90$; 95% CI [−3.85, −1.92]). Importantly, we found significant evidence for topographical content for both tasks in the non-primary ROIs (all $t \geq -3.41$; all $p \leq 0.003$; all $d \geq -0.73$; 95% CI [−1.19, −0.25]; Figure 3B), with a trend found for the face task in the leg ROI ($t_{(21)} = -2.03$; $p = 0.055$; $d = -0.43$; 95% CI [−0.87, 0.01]). These multivariate results reveal that topographical information content about body parts, and the hand in particular, can be observed throughout the homunculus.

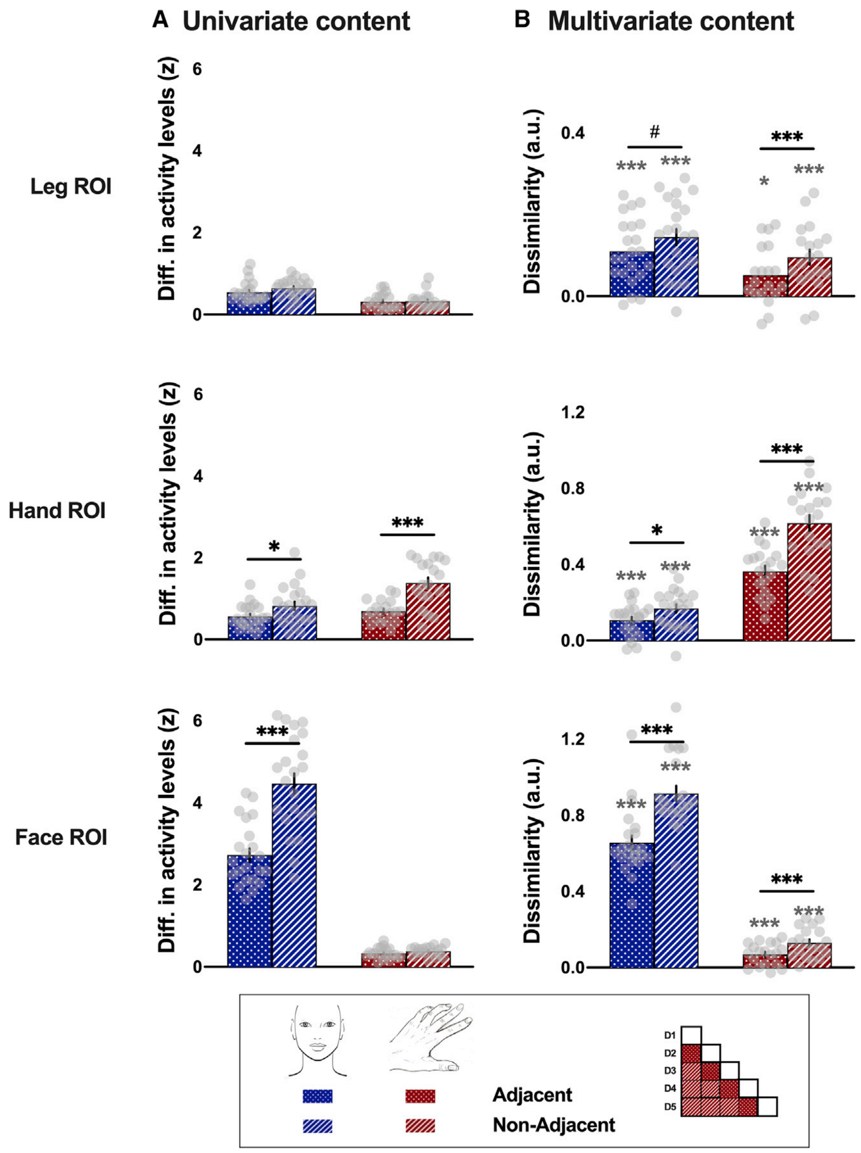

Figure 3. Univariate and multivariate topographic content related to body subparts across S1 homunculus for face and finger tasks

(A) Univariate topographic content defined as the absolute difference between activity levels evoked by adjacent (dotted bars) and non-adjacent (hatched bars) subparts in the different ROIs for the face task (blue) and the finger task (red).

(B) Multivariate topographic content measured by the cross-validated representational dissimilarity (a.u.) between activity patterns evoked by adjacent (dotted bars) and non-adjacent (hatched bars) subparts in the different ROIs for the face task (blue) and the finger task (red).

Data are represented as mean ± SEM. Gray dots represent individual participants. The matrix in the inset illustrates how adjacent and non-adjacent content is computed using the fingers as an example (D, digit). Black asterisks indicate a significant difference between adjacent and non-adjacent body subparts: *$p < 0.05$; #$p < 0.1$; ***$p < 0.001$. Gray asterisks indicate a significant difference relative to zero: *$p < 0.025$; ***$p < 0.001$.

Two actions done with the same body part can be differentiated in non-primary regions of the homunculus

We then assessed how information from different actions done with a given body part is distributed across S1. For that purpose, we compared the squeeze and push conditions performed with each of three body parts (i.e., feet, hand, and lips; see objects in Figure S1). Alpha was adjusted to 0.017, correcting for the three comparisons across body parts. Activity levels evoked by these actions were significantly different only when performed with the primary body part of each ROI (all $p \leq 0.008$; Figure 4A). No significant differences were observed in non-primary ROIs (all $p \geq 0.050$), except for a trend for feet movements in the hand ROI ($t_{(21)} = -2.55$; $p = 0.019$; Figure 4A). However, multivariate representational dissimilarities between the two actions were significantly greater than zero not only in primary ROIs (all $p < 0.001$; all $d \geq 1.00$; 95% CI [1.00, ∞]) but also in non-primary ROIs for hand and feet movements (all $p \leq 0.002$, all $d \geq 0.69$, 95% CI [0.29, ∞]; except for a trend for feet movements in face ROI: $t_{(21)} = 2.09$, $p = 0.024$, $d = 0.45$, 95% CI [0.07, ∞]; Figure 4B). These results suggest that action-related information content from the hand and the feet seems to be distributed across the homunculus (see STAR Methods for potential explanations for the lack of lips information).

Two actions done with the same body part can be differentiated throughout BA3b

Finally, we investigated the profile of action-related information content specifically in BA3b, known to show the greatest level of selectivity in S1 (Powell and Mountcastle, 1959; Martuzzi et al., 2014; Schellekens et al., 2021), alongside the univariate activity levels used to classically determine body maps (Figure 5). To this end, BA3b's strip was segmented into 29 bands of equal height (Figure 5A) that were then used to calculate activity levels and multivariate dissimilarities at the individual level. Consistent with our previous ROI selectivity analysis, the highest activity levels for each body part, based on univariate analysis, lay within their independently defined primary ROI (gray shades in Figure 5B). As we found before, multivariate dissimilarities were qualitatively apparent beyond the regions where activity levels can be observed (Figure 5C). To reduce the number of comparisons, the peak(s) dissimilarity between two different movements with each primary body part (dotted black lines in Figure 5C) was used to test whether action dissimilarities obtained in the

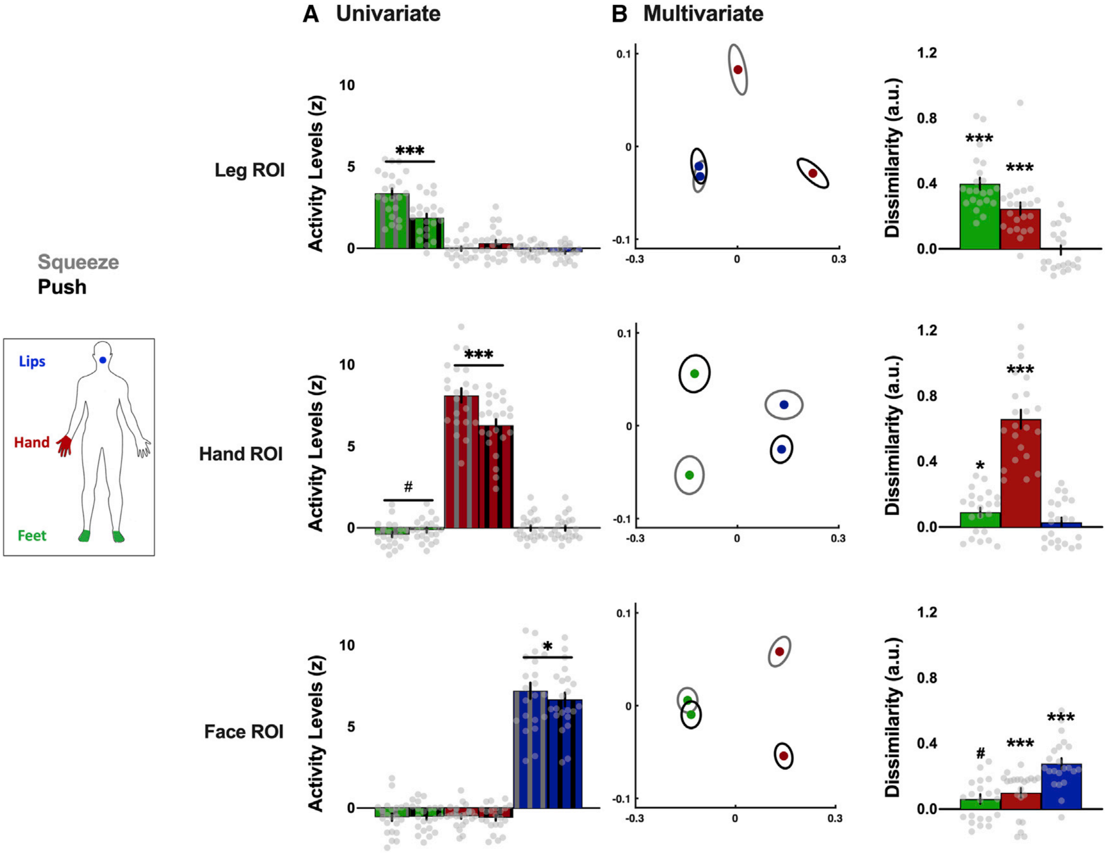

Figure 4. Univariate and multivariate content related to different actions performed with a given body part across S1 homunculus

(A) Univariate activity levels (versus rest) for the two actions (gray hatched, squeeze; black hatched, push) performed with each body part (green, feet; red, hand; blue, lips) within each ROI.

(B) Multivariate dissimilarities. The left plots are a MDS depiction of the representational dissimilarity between the two actions (gray ellipses, squeeze; black ellipses, push) performed with the non-primary body parts in each ROI (green, feet; red, hand; blue, lips). Ellipses indicate between-participant standard errors. The right histograms show the cross-validated dissimilarity (a.u.) observed for the two actions performed with each body part in each ROI (green, feet; red, hand; blue, lips).

Data are represented as mean ± SEM. Gray dots represent individual participants. *p < 0.017; #p < 0.033; ***p < 0.001. See Figure S4 for similar analysis in the non-dominant hemisphere.

corresponding BA3b band using the other non-primary body parts were significantly greater than zero. Alpha was corrected to 0.025 to account for the two comparisons for the two non-primary body parts. For instance, while feet activity levels were observed solely within the first few medial bands of BA3b, dissimilarities between the two actions performed with the feet were significantly greater than zero at the two peaks observed for the hand (both $z \geq 208.00$; both $p \leq 0.003$; both $d \geq 0.64$; 95% CI [0.35, ∞]) but also at the peak observed for the lips ($t_{(21)} = 3.35$; p = 0.002; $d = 0.71$; 95% CI [0.31, ∞]). Similarly, dissimilarities between the two actions performed with the hand were significantly greater than zero at the peaks observed for both the feet and lips (both $t \geq 2.48$; both $p \leq 0.011$; both $d \geq 0.53$; 95% CI [0.15, ∞]). These results emphasize the availability of body part information across S1. Therefore, we find that the information content about body part actions is much more widely distributed then can be inferred by delineating the univariate selectivity profiles.

DISCUSSION

Due to its highly selective profile, conventional mapping procedures providing a "parcellated"—all or nothing (i.e., winner takes all)—view over S1 have dominated our conceptualization of its functional organization (Roux et al., 2018; Willoughby et al., 2021). Consequently, alteration of map boundaries have been commonly interpreted as cortical reorganization, with the limitations previously discussed (Muret and Makin, 2021). Using conventional univariate analyses, together with multivariate RSA, we investigated the distribution of information content underlying S1 topographic organization. We found that S1 contained significant task-relevant information content beyond the primary region of a given body part, as defined by conventional mapping criteria. Even though, as expected from somatotopy, information content was more pronounced in primary regions, cortically distant body parts but also body subparts (e.g., fingers) could be consistently discriminated throughout S1. Perhaps most

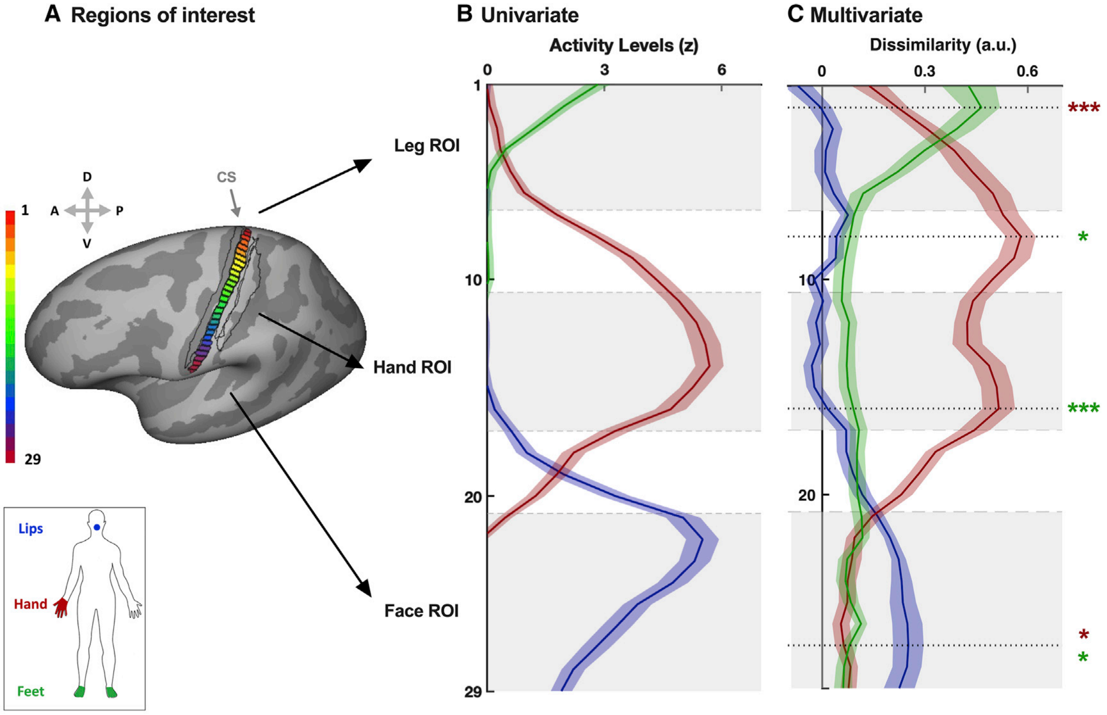

Figure 5. Regions of interest, selectivity, and multivariate information content related to the two actions across BA3b's strip

(A) Illustration of the segmentation of BA3b's strip into 29 bands of similar height (i.e., 2.0911 mm). The black outlines represent the surrounding S1 Brodmann areas, respectively, BA3a, BA1, and BA2 (from left to right), based on a probabilistic atlas. The color code represents the band number (1–29). CS, central sulcus.
(B) Univariate activity levels (versus rest) observed for the three body parts throughout BA3b strip (green, feet; red, hand; blue, lips).
(C) Multivariate cross-validated dissimilarities (a.u.) observed between the two actions (i.e., squeeze and push) for the three pairs of body parts throughout BA3b strip (green, feet; red, hand; blue, lips). The peak dissimilarity for each primary body part (dotted black lines) was used to test whether dissimilarities obtained in the corresponding band of BA3b for the other non-primary body parts were significantly greater than zero; *$p < 0.025$; ***$p < 0.001$.
Data are represented as mean (curves) ± SEM (shades around each curve). Gray shades in the background of each plot represent the location of our leg, hand, and face ROIs.

strikingly, different actions performed with the hand or the feet could be differentiated at remote extremities of the homunculus. Overall, our results suggest a widespread distribution of information content across the S1 homunculus that goes beyond what can be expected from its selectivity profile and emphasizes the need to consider S1 more as a whole than as a patchwork of independent body maps. Our results also stress the need to further investigate the functional relevance of the distributed information and its potential for rehabilitation, augmentation, or brain-machine interfaces.

The widespread availability of body part information was further confirmed by focusing the analysis on discriminating different actions done with the same body part along the most topographically organized sub-region of S1, BA3b. This analysis revealed that, while information content was the highest in primary regions and substantially reduced in non-primary regions, a significant amount of information (related to body parts and actions) persisted throughout BA3b. In particular, it is interesting to note that conventional functional "boundaries" between body maps, as defined by contrasting univariate activity (e.g., between hand and face; Kuehn et al., 2017), did not seem to abruptly disrupt the distribution of information content. Moreover, our data suggest that functional activity and information content is not restricted or compartmentalized by anatomical septa (Fang et al., 2002; Kuehn et al., 2017). Finally, when present (i.e., for hand and feet), information content did not seem to decrease linearly with cortical distance (e.g., feet dissimilarities appeared relatively constant outside the feet region; see Figure 5). This observation might indicate that the distributed information reported here is more likely to arise from thalamo-cortical projections (Rausell et al., 1998) than from horizontal cortico-cortical connections within S1 (Négyessy et al., 2013). However, horizontal connections could still contribute to some extent (Johnson and Frostig, 2015). In addition, potential top-down modulation from higher order regions that are integrating signal across body parts could also contribute to the distribution of information across S1 (Cerkevich and Kaas, 2019). Further work would be required to tease apart these potential origins. Altogether, the widespread distribution of information content even within BA3b suggests that the common spatial definition of body part representation within S1 may only reflect one (if dominant) aspect of S1 organization.

In contrast to the hand and feet, lips' action-related information content appeared to be more restricted to the face region (see Figure 5). This result contrasts with the widespread topographical content we found throughout the homunculus for the

face subparts (see Figure 3). The lack of action-related information for the lips could arise from a lower extent and richness of sensory feedbacks since, contrary to the other body parts, most participants did not manipulate an object during the two lips actions (see STAR Methods). Alternatively, the lips could require less coordination with other body parts, resulting in less representational overlap. Specifically, when coordinating actions, the face is most often the recipient of targeted actions, but not the supplier, contrary to the limbs. This idea is also compatible with the observation of higher resting-state functional connectivity between the hand and feet regions than between the face and the other regions in BA3b (Thomas et al., 2021).

In our view, the observation of widespread information content for body (sub)parts and actions across S1 is not surprising since some extent of distributed tactile information in S1 was already documented by Penfield and Boldrey, based on electrical stimulation (1937). We also do not believe these findings could be discarded as an fMRI artifact due to contribution of blood-stealing effects (Woolsey et al., 1996; Harel et al., 2002; Devor et al., 2005), where local increase in blood flow also results in a decreased blood flow in the immediate surrounding areas. This is because we observe abundant information content remotely from the primary region, which likely extends beyond the spatial scale of these effects (Woolsey et al., 1996; Devor et al., 2005). In contrast, the information we detect is likely related to patterns of negative BOLD responses previously observed in S1 (Tal et al., 2017), responses that were linked to neuronal activity rather than blood-flow-stealing effects (Shmuel et al., 2006; Schridde et al., 2008; Mullinger et al., 2014). We add to this body of previous findings by demonstrating that body part information can be found across the homunculus and is not restricted by functional boundaries or affected by cortical proximity outside the primary region. Nevertheless, we do find that qualitatively greater sharing of information exists between the hand and the feet, as well as between the hand and the mouth. This could be driven by the topographic relationship of the primary regions (where the hand is roughly equidistant between the feet and the mouth). Alternatively, this might reflect differences in the functional usage of body parts: the hands and upper limbs are our main effector to reach and interact with other body parts and for functional coordination with the mouth and the lower limbs for goal-directed behaviors (e.g., for feeding and locomotion and balancing, respectively).

It could be argued that the use of active paradigms in the present study have resulted in more distributed information than would be obtained with a passive tactile paradigm. For example, the distributed information content reported here might result from S1's role in supporting selective movement generation. It has been suggested that latent activity in M1 could contribute to inhibit movement in other body parts not involved in the task (Zeharia et al., 2012) or to afford better motor coordination across body parts (Graziano et al., 2002). Similarly, latent activity in S1 could serve a role for predicting and encoding whole-body sensory feedback expected and perceived during actions involving multiple body parts. Therefore, a passive stimulation of body parts may not necessarily produce widespread information content in S1. While this needs to be investigated, it is important to note that recent studies using similar multivariate analyses in the hand region showed that the representational multivariate structure, as well as the univariate topographic map, were comparable between active paradigm and passively applied tactile stimulation to individual fingers (Berlot et al., 2019; Sanders et al., 2019). Comparable discriminability of hand gestures or postures was also previously reported in humans using electrocorticography, with similar decoding abilities in S1 (Chestek et al., 2013; Branco et al., 2017; Li et al., 2017) and in M1 (Branco et al., 2017). Conversely, recent studies showed that finger movements and effector information can be decoded in S1 during motor planning, well before movement execution (Ariani et al., 2020; Gale et al., 2021). Thus, an active paradigm allows us to take full advantage of sensorimotor information, relevant for motor planning (Sun et al., 2015) and encompass signal arising from the efference copy (London and Miller, 2013). Other recent studies indicate that information content in S1 can be probed even in the absence of sensorimotor inputs. For example, visual observation of roughness exploration with a finger (Kim et al., 2018) or even imagined tactile percepts (Bashford et al., 2021) could be decoded in S1. As such, while the specific contribution of the abundance of tactile, proprioceptive, and even cognitive (Meftah et al., 2002) inputs provided by an active paradigm needs to be further dissociated, all of it is crucial for S1 function. Therefore, the use of an active paradigm is arguably more appropriate to investigate ecological representational motifs.

Microstimulation in the S1 hand region most often elicits sensations on the patients' hand (Flesher et al., 2016; Armenta Salas et al., 2018; Chandrasekaran et al., 2021), and stroke in the M1 hand region results in motor impairment of the hand (Darling et al., 2016). Such selectivity in the sensations induced by focal S1 microstimulation showcases the potential lack of functional relevance of the content reported here. In line with these studies, it is important to clarify that we do not negate the notion of a primary function for a given S1 region. But it is also worth noting that exogeneous stimulation of S1 is an artificial procedure (eliciting unnatural percepts) that is unlikely to tap into the full ecological functioning of S1 and that perceptual assessments in these clinical observations in humans are relatively crude (Richards et al., 2015). For instance, potential (albeit weaker) sensations to other body parts have not been investigated with the refined psychophysical tests that would be required to detect the more subtle effects that we uncover. As such, these clinical reports are not incompatible with the alternative eventuality that the latent activity, which comprises the information content we are reporting, might also be functionally relevant. For example, multiple reports of "referred sensations" across body parts have been documented, either under lab-based manipulations (Badde et al., 2019; Amoruso et al., 2021) or spontaneously (e.g., Katz and Melzack, 1987; Ramachandran et al., 1992; Borsook et al., 1998; Moore et al., 2000; McCabe et al., 2003; Soler et al., 2010). Distributing the content of information throughout S1 could allow for an increased number of combinations and patterns throughout body parts (Hoffmann et al., 2018), which might be more ecologically relevant, considering that we rarely use body parts independently from each other. In other words, this distributed information could provide a way to support

CellPress
OPEN ACCESS

coordinated movements between body parts and to give context to their resulting sensory inputs in a coherent manner. Even if the distributed information underlying the traditional homunculus may not serve a functional role under normal circumstances, it could represent an underlying "scaffolding" for plasticity to take place, such as following congenital (Hahamy et al., 2017) or acquired (Pons et al., 1991) deprivation. For example, latent activity could be harnessed to restore the deprived primary function by potentiating any residual, now latent, activity (Qi et al., 2014). This idea could open perspectives for rehabilitative strategies.

Limitations of the study

One important consideration is that we may have found this distributed information because multivariate techniques are too sensitive. For example, the existence of distributed tactile information outside of the sensorimotor cortex was recently detected in neurons as far as in the rodent primary visual cortex (Enander et al., 2019). As such, being able to decode (or differentiate) across conditions does not necessarily mean the brain is actually using this information (i.e., functionally represented). Even if not functionally valuable, this information could be exploited for brain-machine interfaces (Flesher et al., 2021), where specific parts of the homunculus might not be as directly accessible (e.g., the medial foot region). Finally, the distribution of information across the homunculus and redundancy of information that it might entail could prove particularly useful for solving the issue of "resource reallocation" that augmentation techniques are currently facing (Dominijanni et al., 2021).

To conclude, our results suggest that information in S1 might be a lot more distributed than selectivity profiles and winner-takes-all mapping approaches lead us to presume. While the functional consequences of this widespread information need to be further investigated, it reveals yet unexplored underlying information contents. This abundant information could be harnessed for better functional integration across body parts but also between brain and artificial body parts for rehabilitation, restoration, and augmentation purposes.

STAR★METHODS

Detailed methods are provided in the online version of this paper and include the following:

- KEY RESOURCES TABLE
- RESOURCE AVAILABILITY
  - Lead contact
  - Materials availability
  - Data and code availability
- EXPERIMENTAL MODEL AND SUBJECT DETAILS
- METHOD DETAILS
  - Scanning procedures
  - Body and face tasks
  - Finger task
  - Functional localiser
  - MRI data acquisition
- QUANTIFICATION AND STATISTICAL ANALYSIS
  - fMRI pre-processing
  - fMRI low-level analysis
  - Definition of regions of interest (ROIs)
  - Univariate analysis
  - Multivariate representational similarity analysis
  - BA3b analysis
  - Statistical analysis
  - Group-level ROI visualisations

SUPPLEMENTAL INFORMATION

Supplemental information can be found online at https://doi.org/10.1016/j.celrep.2022.110523.

ACKNOWLEDGMENTS

This work was supported by an ERC Starting Grant (715022 EmbodiedTech) and a Wellcome Trust Senior Research Fellowship (215575/Z/19/Z), awarded to T.R.M. We thank Arabella Bouzigues and Maria Kromm for their substantial help in terms of recruitment and data collection; we also thank Adriana Zainurin, Esther Teo, Christine Tan, and Mathew Kollamkulam for help with data collection.

AUTHOR CONTRIBUTIONS

D.M. and T.R.M. conceived the study. D.C. designed the objects. D.M. collected the body and face datasets, and P.K. and D.C. collected the finger dataset. V.R. pre-processed the face dataset, D.M. pre-processed the body dataset and analyzed the body and face datasets, and P.K. pre-processed and analyzed the finger dataset. D.M. and T.R.M. wrote the manuscript with input from all co-authors. T.R.M. secured funding.

DECLARATION OF INTERESTS

The authors declare no competing interests.

INCLUSION AND DIVERSITY

We worked to ensure gender balance in the recruitment of human subjects. One or more of the authors of this paper self-identifies as a member of the LGBTQ+ community.

Received: September 20, 2021
Revised: December 10, 2021
Accepted: February 21, 2022
Published: March 15, 2022

REFERENCES

Amoruso, E., Terhune, D.B., Kromm, M., Kirker, S., Muret, D., and Makin, T.R. (2021). Reassessing referred sensations following peripheral deafferentation and the role of cortical reorganisation. Preprint at medRxiv. https://doi.org/10.1101/2021.12.08.21267128.

Ariani, G., Pruszynski, J.A., and Diedrichsen, J. (2020). Motor Planning Brings Human Primary Somatosensory Cortex into Movement-specific Preparatory States. Preprint at bioRxiv. https://doi.org/10.1101/2020.12.17.423254.

Armenta Salas, M., Bashford, L., Kellis, S., Jafari, M., Jo, H., Kramer, D., Shanfield, K., Pejsa, K., Lee, B., Liu, C.Y., et al. (2018). Proprioceptive and cutaneous sensations in humans elicited by intracortical microstimulation. Elife *7*, 1–11. https://doi.org/10.7554/eLife.32904.

Auffret, M., Ravano, V.L., Rossi, G.M.C., Hankov, N., Petersen, M.F.A., and Petersen, C.C.H. (2018). Optogenetic Stimulation of Cortex to Map Evoked Whisker Movements in Awake Head-Restrained Mice. Neuroscience *368*, 199–213. https://doi.org/10.1016/j.neuroscience.2017.04.004.

Badde, S., Röder, B., and Heed, T. (2019). Feeling a touch to the hand on the foot. Curr. Biol. *29*, 1491–1497.e4. https://doi.org/10.1016/j.cub.2019.02.060.

Baldwin, M.K., Cooke, D.F., and Krubitzer, L. (2017). Intracortical microstimulation maps of motor, somatosensory, and posterior parietal cortex in tree shrews (Tupaia belangeri) reveal complex movement representations. Cereb. Cortex *27*, 1439–1456. https://doi.org/10.1093/cercor/bhv329.

Baldwin, M.K.L., Cooke, D.F., Goldring, A.B., and Krubitzer, L. (2018). Representations of fine digit movements in posterior and anterior parietal cortex revealed using long-train intracortical microstimulation in macaque monkeys. Cereb. Cortex *28*, 4244–4263. https://doi.org/10.1093/cercor/bhx279.

Bashford, L., Rosenthal, I., Kellis, S., Pejsa, K., Kramer, D., Lee, B., Liu, C., and Andersen, R.A. (2021). The neurophysiological representation of imagined somatosensory percepts in human cortex. J. Neurosci. *41*, 2177–2185. https://doi.org/10.1523/jneurosci.2460-20.2021.

Bensmaia, S.J., and Miller, L.E. (2014). Restoring sensorimotor function through intracortical interfaces: progress and looming challenges. Nat. Rev. Neurosci. *15*, 313–325. https://doi.org/10.1038/nrn3724.

Berlot, E., Prichard, G., O'Reilly, J., Ejaz, N., and Diedrichsen, J. (2019). Ipsilateral finger representations in the sensorimotor cortex are driven by active movement processes, not passive sensory input. J. Neurophysiol. *121*, 418–426. https://doi.org/10.1152/jn.00439.2018.

Borsook, D., Becerra, L., Fishman, S., Edwards, A., Jennings, C.L., Stojanovic, M., Papinicolas, L., Ramachandran, V.S., Gonzalez, R.G., and Breiter, H. (1998). Acute plasticity in the human somatosensory cortex following amputation. Neuroreport *9*, 1013–1017.

Branco, M.P., Freudenburg, Z.V., Aarnoutse, E.J., Bleichner, M.G., Vansteensel, M.J., and Ramsey, N.F. (2017). Decoding Hand Gestures from Primary Somatosensory Cortex Using High-Density ECoG. Neuroimage *147*, 130–142. https://doi.org/10.1016/j.neuroimage.2016.12.004.

Catani, M., Dell'acqua, F., Vergani, F., Malik, F., Hodge, H., Roy, P., Valabregue, R., and Thiebaut de Schotten, M. (2012). Short frontal lobe connections of the human brain. Cortex *48*, 273–291. https://doi.org/10.1016/j.cortex.2011.12.001.

Cerkevich, C.M., and Kaas, J.H. (2019). Corticocortical projections to area 1 in squirrel monkeys (Saimiri sciureus). Eur. J. Neurosci. *49*, 1024–1040. https://doi.org/10.1111/ejn.13884.

Chandrasekaran, S., Bickel, S., Herrero, J.L., Kim, J.W., Markowitz, N., Espinal, E., Bhagat, N.A., Ramdeo, R., Xu, J., Glasser, M.F., et al. (2021). Evoking highly focal percepts in the fingertips through targeted stimulation of sulcal regions of the brain for sensory restoration. Brain Stimul. *14*, 1184–1196. https://doi.org/10.1016/j.brs.2021.07.009.

Chestek, C.A., Gilja, V., Blabe, C.H., Foster, B.L., Shenoy, K.V., Parvizi, J., and Henderson, J.M. (2013). Hand posture classification using electrocorticography signals in the gamma band over human sensorimotor brain areas. J. Neural. Eng. *10*, 026002. https://doi.org/10.1088/1741-2560/10/2/026002.

Cohen, J. (1988). Statistical Power Analysis for the Behavioral Sciences (Academic), p. 54.

Cunningham, D.A., Machado, A., Yue, G.H., Carey, J.R., and Plow, E.B. (2013). Functional somatotopy revealed across multiple cortical regions using a model of complex motor task. Brain Res. *1531*, 25–36. https://doi.org/10.1016/j.brainres.2013.07.050.

Cybulska-Klosowicz, A., Tremblay, F., Jiang, W., Bourgeon, S., Meftah, E.M., and Chapman, C.E. (2020). Differential effects of the mode of touch, active and passive, on experience-driven plasticity in the S1 cutaneous digit representation of adult macaque monkeys. J. Neurophysiol. *123*, 1072–1089. https://doi.org/10.1152/jn.00014.2019.

Dale, A.M., Fischl, B., and Sereno, M.I. (1999). Cortical surface-based analysis. I. Segmentation and surface reconstruction. Neuroimage *9*, 179–194. https://doi.org/10.1006/nimg.1998.0395.

Darling, W.G., Pizzimenti, M.A., Rotella, D.L., Hynes, S.M., Ge, J., Stilwell-Morecraft, K., and Morecraft, R.J. (2016). Sensorimotor cortex injury effects on recovery of contralesional dexterous movements in macaca mulatta. Exp. Neurol. *281*, 37–52. https://doi.org/10.1016/j.expneurol.2016.04.004.

Dempsey-Jones, H., Themistocleous, A.C., Carone, D., Ng, T.W.C., Harrar, V., and Makin, T.R. (2019). Blocking tactile input to one finger using anaesthetic enhances touch perception and learning in other fingers. J. Exp. Psychol. Gen. *148*, 713–727. https://doi.org/10.1037/xge0000514.

Devor, A., Ulbert, I., Dunn, A.K., Narayanan, S.N., Jones, S.R., Andermann, M.L., Boas, D.A., and Dale, A.M. (2005). Coupling of the cortical hemodynamic response to cortical and thalamic neuronal activity. Proc. Natl. Acad. Sci. U S A *102*, 3822–3877. https://doi.org/10.1073/pnas.0407789102.

Diedrichsen, J., Provost, S., and Zareamoghaddam, H. (2016). On the Distribution of Cross-Validated Mahalanobis Distances. Preprint at arXiv. 1607.01371.

Dominijanni, G., Shokur, S., Salvietti, G., Buehler, S., Palmerini, E., Rossi, S., De Vignemont, F., D'Avella, A., Makin, T.R., Prattichizzo, D., and Micera, S. (2021). Enhancing human bodies with extra robotic arms and fingers: the neural resource allocation problem. Preprint at arxiv. 2103.17252.

Ejaz, N., Hamada, M., and Diedrichsen, J. (2015). Hand use predicts the structure of representations in sensorimotor cortex. Nat. Neurosci. *18*, 1034–1040. https://doi.org/10.1038/nn.4038.

Enander, J.M.D., Spanne, A., Mazzoni, A., Bengtsson, F., Oddo, C.M., and Jörntell, H. (2019). Ubiquitous neocortical decoding of tactile input patterns. Front. Cell Neurosci. *13*, 1–17. https://doi.org/10.3389/fncel.2019.00140.

Enander, J.M.D., and Jörntell, H. (2019). Somatosensory cortical neurons decode tactile input patterns and location from both dominant and non-dominant digits. Cell Rep. *26*, 3551–3560.e4. https://doi.org/10.1016/j.celrep.2019.02.099.

Fang, P.C., Jain, N., and Kaas, J.H. (2002). Few intrinsic connections cross the hand-face border of area 3b of New World monkeys. J. Comp. Neurol. *454*, 310–319.

Ferrier, D. (1873). Experimental researches in cerebral physiology and pathology. Br. Med. J. *1*, 457.

Fischl, B., Liu, A., and Dale, A.M. (2001). Automated manifold surgery: constructing geometrically accurate and topologically correct models of the human cerebral cortex. IEEE Trans. Med. Imaging *20*, 70–80. https://doi.org/10.1109/42.906426.

Flesher, S.N., Downey, J.E., Weiss, J.M., Hughes, C.L., Herrera, A.J., Tyler-Kabara, E.C., Boninger, M.L., Collinger, J.L., and Gaunt, R.A. (2021). A brain-computer interface that evokes tactile sensations improves robotic arm control. Science *372*, 831–836.

Flesher, S.N., Collinger, J.L., Foldes, S.T., Weiss, J.M., Downey, J.E., Tyler-Kabara, E.C., Bensmaia, S.J., Schwartz, A.B., Boninger, M.L., and Gaunt, R.A. (2016). Intracortical microstimulation of human somatosensory cortex. Sci. Transl. Med. *8*, 361ra141–ra211. https://doi.org/10.1126/scitranslmed.aaf8083.

Friston, K.J., Fletcher, P., Josephs, O., Holmes, A., Rugg, M.D., and Turner, R. (1998). Event-related fMRI: characterizing differential responses. Neuroimage *7*, 30–40. https://doi.org/10.1006/nimg.1997.0306.

Fritsch, G.T., and Hitzig, E. (1870). On the electrical excitability of the cerebrum. International Classics in Epilepsy and Behavior *15*, P123–P130.

Gale, D.J., Flanagan, J.R., and Gallivan, J.P. (2021). Human somatosensory cortex is modulated during motor planning. J. Neurosci. *41*, 5909–5922. https://doi.org/10.1523/jneurosci.0342-21.2021.

Germann, J., Chakravarty, M.M., Collins, D.L., and Petrides, M. (2020). Tight coupling between morphological features of the central sulcus and somatomotor body representations: a combined anatomical and functional mri study. Cereb Cortex *30*, 1843–1854. https://doi.org/10.1093/cercor/bhz208.

Glasser, M.F., Coalson, T.S., Robinson, E.C., Hacker, C.D., Harwell, J., Yacoub, E., Ugurbil, K., Andersson, J., Beckmann, C.F., Jenkinson, M., Smith, S.M., and Van Essen, D.C. (2016). A multi-modal parcellation of human cerebral cortex. Nature *536*, 171–178.

Graziano, M.S., and Aflalo, T.N. (2007). Mapping behavioral repertoire onto the cortex. Neuron *56*, 239–251. https://doi.org/10.1016/j.neuron.2007.09.013.

Graziano, M.S., Taylor, C.S., and Moore, T. (2002). Complex movements evoked by microstimulation of precentral cortex. Neuron *34*, 841–851. https://doi.org/10.1016/S0896-6273(02)00698-0.

Greve, D.N., and Fischl, B. (2009). Accurate and robust brain image alignment using boundary-based registration. Neuroimage *48*, 63–72. https://doi.org/10.1016/j.neuroimage.2009.06.060.

Hahamy, A., Macdonald, S.N., van den Heiligenberg, F., Kieliba, P., Emir, U., Malach, R., Johansen-Berg, H., Brugger, P., Culham, J.C., and Makin, T.R. (2017). Representation of multiple body parts in the missing-hand territory of congenital one-handers. Curr. Biol. *27*, 1350–1355. https://doi.org/10.1016/j.cub.2017.03.053.

Halley, A.C., Baldwin, M.K.L., Cooke, D.F., Englund, M., and Krubitzer, L. (2020). Distributed motor control of limb movements in rat motor and somatosensory cortex: the sensorimotor amalgam revisited. Cereb. Cortex *30*, 6296–6312. https://doi.org/10.1093/cercor/bhaa186.

Harel, N., Lee, S.P., Nagaoka, T., Kim, D.S., and Kim, S.G. (2002). Origin of negative blood oxygenation level-dependent fMRI signals. J. Cereb. Blood Flow Metab. *22*, 908–917. https://doi.org/10.1097/00004647-200208000-00002.

Hoffmann, M., Straka, I., Vavrečka, F.M., and Metta, G. (2018). Robotic homunculus: learning of artificial skin representation in a humanoid robot motivated by primary somatosensory cortex. IEEE Trans. Cogn. Dev. Syst. *10*, 163–176. https://doi.org/10.1109/TCDS.2017.2649225.

Huber, L., Finn, E.S., Handwerker, D.A., Bönstrup, M., Glen, D.R., Kashyap, S., Ivanov, D., Petridou, N., Marrett, S., Goense, J., et al. (2020). Sub-millimeter fmri reveals multiple topographical digit representations that form action maps in human motor cortex. Neuroimage *208*, 116463. https://doi.org/10.1016/j.neuroimage.2019.116463.

Jenkinson, M., Bannister, P., Brady, M., and Smith, S. (2002). Improved optimization for the robust and accurate linear registration and motion correction of brain images. Neuroimage *17*, 825–841. https://doi.org/10.1006/nimg.2002.1132.

Jenkinson, M., Beckmann, C.F., Behrens, T.E., Woolrich, M.W., and Smith, S.M. (2012). FSL. Neuroimage *62*, 782–790. https://doi.org/10.1016/j.neuroimage.2011.09.015.

Johnson, B.A., and Frostig, R.D. (2015). Long, intrinsic horizontal axons radiating through and beyond rat barrel cortex have spatial distributions similar to horizontal spreads of activity evoked by whisker stimulation. Brain Struct. Funct. *221*. https://doi.org/10.1007/s00429-015-1123-7.

Kaas, J.H., Nelson, R.J., Sur, M., Lin, C.S., and Merzenich, M.M. (1979). Multiple representations of the body within the primary somatosensory cortex of primates. Science *204*, 521–523. https://doi.org/10.1126/science.107591.

Katz, J., and Melzack, R. (1987). Referred sensations in chronic pain patients. Pain *28*, 51–59. https://doi.org/10.1016/0304-3959(87)91059-1.

Kieliba, P., Clode, D., Maimon-Mor, R.O., and Makin, T.R. (2021). Robotic hand augmentation drives changes in neural body representation. Sci. Robotics *6*, eabd7935. https://doi.org/10.1126/scirobotics.abd7935.

Kim, J., Bülthoff, I., and Bülthoff, H.H. (2018). Decoding visual roughness perception: an fMRI study. Somatosens Mot Res *35*, 212–217. https://doi.org/10.1080/08990220.2018.1527761.

Kuehn, E., Dinse, J., Jakobsen, E., Long, X., Schäfer, A., Bazin, P.L., Villringer, A., Sereno, M.I., and Margulies, D.S. (2017). Body topography parcellates human sensory and motor cortex. Cereb. Cortex *27*, 3790–3805. https://doi.org/10.1093/cercor/bhx026.

Kumar, N., Manning, T.F., and Ostry, D.J. (2019). Somatosensory cortex participates in the consolidation of human motor memory. PLoS Biol. *17*, e3000469–21. https://doi.org/10.1371/journal.pbio.3000469.

Li, Y., Zhang, S., Jin, Y., Cai, B., Controzzi, M., Zhu, J., Zhang, J., and Zheng, X. (2017). Gesture decoding using ECoG signals from human sensorimotor cortex: a pilot study. Behav. Neurol. *2017*, 1–12. https://doi.org/10.1155/2017/3435686.

London, B.M., and Miller, L.E. (2013). Responses of somatosensory area 2 neurons to actively and passively generated limb movements. J. Neurophysiol. *109*, 1505–1513. https://doi.org/10.1152/jn.00372.2012.

Martuzzi, R., van der Zwaag, W., Farthouat, J., Gruetter, R., and Blanke, O. (2014). Human finger somatotopy in areas 3b, 1, and 2: a 7T fMRI study using a natural stimulus. Hum. Brain Mapp. *35*, 213–226. https://doi.org/10.1002/hbm.22172.

Matyas, F., Sreenivasan, V., Marbach, F., Wacongne, C., Barsy, B., Mateo, C., Aronoff, R., and Petersen, C.C. (2010). Motor control by sensory cortex. Science *330*, 1240–1243.

Mayer, A., Baldwin, M.K.L., Cooke, D.F., Lima, B.R., Padberg, J., Lewenfus, G., Franca, J.G., and Krubitzer, L. (2019). The multiple representations of complex digit movements in primary motor cortex form the building blocks for complex grip types in capuchin monkeys. J. Neurosci. *39*, 6684–6695. https://doi.org/10.1523/JNEUROSCI.0556-19.2019.

McCabe, C.S., Haigh, R.C., Halligan, P.W., and Blake, D.R. (2003). Referred sensations in patients with complex regional pain syndrome type 1. Rheumatology (Oxford) *42*, 1067–1073. https://doi.org/10.1093/rheumatology/keg298.

Meftah, el-M, Shenasa, J., and Chapman, C.E. (2002). Effects of a cross-modal manipulation of attention on somatosensory cortical neuronal responses to tactile stimuli in the monkey. J. Neurophysiol. *88*, 3133–3149. https://doi.org/10.1152/jn.00121.2002.

Merzenich, M.M., Kaas, J.H., Sur, M., and Lin, C.S. (1978). Double representation of the body surface within cytoarchitectonic areas 3b and 1 in "SI" in the Owl monkey (Aotus trivirgatus). J. Comp. Neurol. *181*, 41–73.

Moore, C.I., Stern, C.E., Dunbar, C., Kostyk, S.K., Gehi, A., and Corkin, S. (2000). Referred phantom sensations and cortical reorganization after spinal cord injury in humans. Proc. Natl. Acad. Sci. U S A *97*, 14703–14708. https://doi.org/10.1073/pnas.250348997.

Mullinger, K.J., Mayhew, S.D., Bagshaw, A.P., Bowtell, R., and Francis, S.T. (2014). Evidence that the Negative BOLD Response Is Neuronal in Origin: A Simultaneous EEG-BOLD-CBF Study in Humans. NeuroImage *94*, 263–274. https://doi.org/10.1016/j.neuroimage.2014.02.029.

Muret, D., and Makin, T.R. (2021). The homeostatic homunculus: rethinking deprivation-triggered reorganisation. Curr. Opin. Neurobiol. *67*, 115–122. https://doi.org/10.1016/j.conb.2020.08.008.

Nakamura, A., Yamada, T., Goto, A., Kato, T., Ito, K., Abe, Y., Kachi, T., and Kakigi, R. (1998). Somatosensory homunculus as drawn by MEG. Neuroimage *7*, 377–386.

Négyessy, L., Pálfi, E., Ashaber, M., Palmer, C., Jákli, B., Friedman, R.M., Chen, L.M., and Roe, A.W. (2013). Intrinsic horizontal connections process global tactile features in the primary somatosensory cortex: neuroanatomical evidence. J. Comp. Neurol. *521*, 2798–2817. https://doi.org/10.1002/cne.23317.

Ngo, G.N., Ngo, G.N., Haak, K.V., Beckmann, C.F., and Menon, R.S. (2021). Mesoscale hierarchical organization of primary somatosensory cortex captured by resting-state-fMRI in humans. Neuroimage *235*, 118031. https://doi.org/10.1016/j.neuroimage.2021.118031.

Nili, H., Wingfield, C., Walther, A., Su, L., Marslen-Wilson, W., and Kriegeskorte, N. (2014). A toolbox for representational similarity analysis. PLoS Comput. Biol. *10*, e1003553. https://doi.org/10.1371/journal.pcbi.1003553.

Penfield, W., and Boldrey, E. (1937). Somatic motor and sensory representation in the cerebral cortex of man as studied by electrical stimulation. Brain *60*, 389–443.

Pons, T.P., Garraghty, P.E., Ommaya, A.K., Kaas, J.H., Taub, E., and Mishkin, M. (1991). Massive cortical reorganization after sensory deafferentation in adult macaques. Science *252*, 1857–1860.

Powell, T.P., and Mountcastle, V.B. (1959). Some aspects of the functional organization of the cortex of the postcentral gyrus of the monkey: a correlation of findings obtained in a single unit analysis with cytoarchitecture. Bull. Johns Hopkins Hosp. *105*, 133–162.

Qi, H.X., Kaas, J.H., and Reed, J.L. (2014). The reactivation of somatosensory cortex and behavioral recovery after sensory loss in mature primates. Front. Syst. Neurosci. *8*, 84–114. https://doi.org/10.3389/fnsys.2014.00084.

Ramachandran, V.S., Rogers-Ramachandran, D., and Stewart, M. (1992). Perceptual correlates of massive cortical reorganization. Science *258*, 1159–1160.

Rausell, E., Bickford, L., Manger, P.R., Woods, T.M., and Jones, E.G. (1998). Extensive divergence and convergence in the thalamocortical projection to monkey somatosensory cortex. J. Neurosci. *18*, 4216–4232.

Richards, C.L., Malouin, F., and Nadeau, S. (2015). Stroke Rehabilitation: Clinical Picture, Assessment, and Therapeutic challenge. Prog. Brain Res. *218*, 253–280. https://doi.org/10.1016/bs.pbr.2015.01.003.

Root, V., Muret, D., Arribas, M., Amoruso, E., Thornton, J., Tarall-Jozwiak, A., Tracey, I., and Makin, T.R. (2021). Investigating Facial Information Content in the Hand Area of Individuals with a Congenital and Acquired Missing Hand. Preprint at bioRxiv. https://doi.org/10.1101/2021.07.05.451126.

Roux, F.E., Djidjeli, I., and Durand, J.B. (2018). Functional architecture of the somatosensory homunculus detected by electrostimulation. J. Physiol. *596*, 941–956. https://doi.org/10.1113/JP275243.

Saadon-Grosman, N., Arzy, S., and Loewenstein, Y. (2020). Hierarchical cortical gradients in somatosensory processing. Neuroimage *222*, 117257. https://doi.org/10.1016/j.neuroimage.2020.117257.

Sanchez Panchuelo, R.M., Ackerley, R., Glover, P.M., Bowtell, R.W., Wessberg, J., Francis, S.T., and McGlone, F. (2016). Mapping quantal touch using 7 tesla functional magnetic resonance imaging and single-unit intraneural microstimulation. Elife *5*, 1–19. https://doi.org/10.7554/eLife.12812.

Sanders, Z.-B., Wesselink, D.B., Dempsey-Jones, H., and Makin, T.R. (2019). Similar Somatotopy for Active and Passive Digit Representation in Primary Somatosensory Cortex. Preprint at bioRxiv. https://doi.org/10.1101/754648.

Schellekens, W., Thio, M., Badde, S., Winawer, J., Ramsey, N., and Petridou, N. (2021). A touch of hierarchy: population receptive fields reveal fingertip integration in Brodmann areas in human primary somatosensory cortex. Brain Struct. Funct. *226*, 2099–2112. https://doi.org/10.1007/s00429-021-02309-5.

Schieber, M.H. (2001). Constraints on somatotopic organization in the primary motor cortex. J. Neurophysiol. *86*, 2125–2143. https://doi.org/10.1152/jn.2001.86.5.2125.

Schridde, U., Khubchandani, M., Motelow, J.E., Sanganahalli, B.G., Hyder, F., and Blumenfeld, H. (2008). Negative BOLD with large increases in neuronal activity. Cereb. Cortex *18*, 1814–1827. https://doi.org/10.1093/cercor/bhm208.

Shmuel, A., Augath, M., Oeltermann, A., and Logothetis, N.K. (2006). Negative functional MRI response correlates with decreases in neuronal activity in monkey visual area V1. Nat. Neurosci. *9*, 569–577. https://doi.org/10.1038/nn1675.

Smith, S.M. (2002). Fast robust automated brain extraction. Hum. Brain Mapp. *17*, 143–155. https://doi.org/10.1002/hbm.10062.

Smith, S.M., Jenkinson, M., Woolrich, M.W., Beckmann, C.F., Behrens, T.E., Johansen-Berg, H., Bannister, P.R., De Luca, M., Drobnjak, I., Flitney, D.E., et al. (2004). Advances in functional and structural MR image analysis and implementation as FSL. Neuroimage *23*, S208–S219. https://doi.org/10.1016/j.neuroimage.2004.07.051.

Soler, M.D., Kumru, H., Vidal, J., Pelayo, R., Tormos, J.M., Fregni, F., Navarro, X., and Pascual-Leone, A. (2010). Referred sensations and neuropathic pain following spinal cord injury. Pain *150*, 192–198. https://doi.org/10.1016/j.pain.2010.04.027.

Sun, F., Zhang, G., Ren, L., Yu, T., Ren, Z., Gao, R., and Zhang, X. (2021). Functional organization of the human primary somatosensory cortex: a stereo-electroencephalography study. Clin. Neurophysiol. *132*, 487–497. https://doi.org/10.1016/j.clinph.2020.11.032.

Sun, H., Blakely, T.M., Darvas, F., Wander, J.D., Johnson, L.A., Su, D.K., Miller, K.J., Fetz, E.E., and Ojemann, J.G. (2015). Sequential activation of premotor, primary somatosensory and primary motor areas in humans during cued finger movements. Clin. Neurophysiol. *126*, 2150–2161. https://doi.org/10.1016/j.clinph.2015.01.005.

Tal, Z., Geva, R., and Amedi, A. (2017). Positive and negative somatotopic BOLD responses in contralateral versus Ipsilateral Penfield homunculus. Cereb. Cortex *27*, 962–980. https://doi.org/10.1093/cercor/bhx024.

Thomas, J., Sharma, D., Mohanta, S., and Jain, N. (2021). Resting-State functional networks of different topographic representations in the somatosensory cortex of macaque monkeys and humans. Neuroimage *228*, 117694. https://doi.org/10.1016/j.neuroimage.2020.117694.

Walther, A., Nili, H., Ejaz, N., Alink, A., Kriegeskorte, N., and Diedrichsen, J. (2016). Reliability of dissimilarity measures for multi-voxel pattern analysis. Neuroimage *137*, 188–200. https://doi.org/10.1016/j.neuroimage.2015.12.012.

Wesselink, D.B., van den Heiligenberg, F.M., Ejaz, N., Dempsey-Jones, H., Cardinali, L., Tarall-Jozwiak, A., Diedrichsen, J., and Makin, T.R. (2019). Obtaining and maintaining cortical hand representation as evidenced from acquired and congenital handlessness. Elife *8*, 1–19. https://doi.org/10.7554/eLife.37227.

Wesselink, D.B., Sanders, Z.B., Edmondson, L.R., Dempsey-Jones, H., Kieliba, P., Kikkert, S., Themistocleous, A.C., Emir, U., Diedrichsen, J., Saal, H.P., and Makin, T.R. (2020). Malleability of the cortical hand map following a single finger nerve block. Preprint at bioRxiv. https://doi.org/10.1101/2020.10.16.338640.

Wesselink, D., and Maimon-Mor, R.O. (2018). RSA toolbox extension for FSL. https://github.com/ronimaimon/rsatoolbox.

Widener, G.L., and Cheney, P.D. (1997). Effects on muscle activity from microstimuli applied to somatosensory and motor cortex during voluntary movement in the monkey. J. Neurophysiol. *77*, 2446–2465. https://doi.org/10.1152/jn.1997.77.5.2446.

Wiech, K., Jbabdi, S., Lin, C.S., Andersson, J., and Tracey, I. (2014). Differential structural and resting state connectivity between insular subdivisions and other pain-related brain regions. Pain *155*, 2047–2055. https://doi.org/10.1016/j.pain.2014.07.009.

Wiestler, T., and Diedrichsen, J. (2013). Skill learning strengthens cortical representations of motor sequences. Elife *2*, e00801–e00820. https://doi.org/10.7554/eLife.00801.

Willoughby, W.R., Thoenes, K., and Bolding, M. (2021). Somatotopic arrangement of the human primary somatosensory cortex derived from functional magnetic resonance imaging. Front. Neurosci. *14*, 1–10. https://doi.org/10.3389/fnins.2020.598482.

Woolsey, T.A., Rovainen, C.M., Cox, S.B., Henegar, M.H., Liang, G.E., Liu, D., Moskalenko, Y.E., Sui, J., and Wei, L. (1996). Neuronal units linked to microvascular modules in cerebral cortex: response elements for imaging the brain. Cereb. Cortex *6*, 647–660. https://doi.org/10.1093/cercor/6.5.647.

Zeharia, N., Hertz, U., Flash, T., and Amedi, A. (2012). Negative blood oxygenation level dependent homunculus and somatotopic information in primary motor cortex and supplementary motor area. Proc. Natl. Acad. Sci. U S A *109*, 18565–18570. https://doi.org/10.1073/pnas.1119125109.

STAR★METHODS

KEY RESOURCES TABLE

REAGENT or RESOURCE	SOURCE	IDENTIFIER
Deposited data		
fMRI data	This paper	https://osf.io/g3y5u/ https://doi.org/10.17605/OSF.IO/G3Y5U
Software and algorithms		
FSL v. 6.00	Smith et al., 2004; Jenkinson et al., 2012	https://fsl.fmrib.ox.ac.uk
Connectome Workbench v. 1.4.2	https://www.humanconnectome.org	https://www.humanconnectome.org
Matlab v. R2016a	Mathworks	https://www.mathworks.com
Freesurfer v. 7.1.1	Dale et al. (1999); Fischl et al. 2001	https://freesurfer.net
JASP v. 0.14	https://github.com/jasp-stats/jasp-desktop/commit/f2cb223fef6b15089fd6e9da54825d370c1ece57	https://jasp-stats.org/

RESOURCE AVAILABILITY

Lead contact
For further information and requests for resources should be directed to and will be fulfilled by the lead contact, Dollyane Muret (d.muret@ucl.ac.uk).

Materials availability
The study did not generate new materials.

Data and code availability
- The data generated during this study will be available from the Open Science Framework upon publication (https://osf.io/g3y5u/).
- This paper does not report original code.
- Any additional information required to reanalyse the data reported in this paper is available from the lead contact upon request.

EXPERIMENTAL MODEL AND SUBJECT DETAILS

Twenty-two healthy volunteers [mean age = 45.55 ± 9.47 (SD) years; 10 women; 6 left-handed] took part in the body and face tasks and a further nineteen healthy volunteers [mean age = 23.16 ± 4.34 (SD) years; 11 women; all right-handed] took part in the finger task. To account for age-related differences, age was added as a covariate in statistical analyses. Participants reported no sensorimotor disorders and had no counterindications for magnetic resonance imaging. All participants gave written informed consent before participating. The protocols were approved by the NHS National Research Ethics Service approval (18/LO/0474) for the body and face tasks and UCL Research Ethics Committee (REC: 12921/001) for the finger task and were performed in accordance with the Declaration of Helsinki. The face and hand datasets were recently used for other purposes (Kieliba et al., 2021; Root et al., 2021).

METHOD DETAILS

Scanning procedures
Each dataset comprised three or four functional task-related block-design runs, a functional localiser, a structural scan and field maps.

Body and face tasks
The two tasks were performed within the same experimental session. Prior to entering the scanner room, participants were thoroughly instructed, and all movements were practiced in front of the experimenter to ensure they were performed correctly. For the body task, participants were instructed to perform one of two actions (i.e., squeeze or push; Figure S1) with one of three different

body parts (i.e., feet, dominant hand and lips), resulting in a total of six conditions. Two additional conditions involving the non-dominant arm were also included but will not be described in the main text since we focus on the hemisphere contralateral to each body part. See supplemental information for similar analyses and results in the non-dominant hemisphere for the body task (Figures S2 and S4). For the face task, participants were instructed to perform one of four movements: raise the eyebrows (i.e., forehead), flare nostrils (i.e., nose), puckering lips (i.e., lips), and tap the tongue to the roof of the mouth (i.e., tongue). Two additional conditions involving the left and right thumbs were also included but will not be further described as they were not included in the main analysis (see Root et al., 2021 for analysis of these conditions).

For both tasks, instructions and pace were provided visually via a screen, resulting in 5 cycles of movement per 8 sec block. In addition, each movement block was repeated 4 times per run, which also comprised 5 blocks of rest used as baseline. Conditions were pseudo-randomly distributed, such that each condition was equally preceded by all other conditions. Three and four functional runs were acquired for the face and body tasks, respectively. To confirm that appropriate movements were made at the instructed times, task performance was visually monitored online.

Finger task

Participants performed an active finger tapping task using a button box. Each finger movement was repeated at 1Hz over a period of 9s per block, with 4 blocks per finger per run in a semi-counterbalanced order and 4 runs in total. Instructions and pace were provided visually. Ten vertical bars, representing the fingers, flashed individually in green at a frequency of 1 Hz. Task performance was monitored online. Two additional conditions involving the feet and lips were also included but will not be further described as they were not included in the main analysis (see Kieliba et al., 2021 for analysis of these conditions).

Functional localiser

Participants were visually instructed to move one of five body parts: right or left hand (open/closing the fingers), right or left toes (wiggling the toes) or lips (puckering the lips). Movements were repeated at a constant instructed pace for a period of 12s, interleaved with 12s of rest. Each condition was repeated 4 times in a pseudo-random order. Here again, participants practiced the movements before entering the scanner and task performance was visually monitored online.

MRI data acquisition

MRI images were acquired using a 3T Prisma MRI scanner (Siemens, Erlangen, Germany) with a 32-channel head coil. Functional data were obtained using a multiband T2*-weighted pulse sequence with a between-slice acceleration factor of 4 and no in-slice acceleration. The following acquisition parameters were used: TR = 1450 ms; TE = 35 ms; flip angle =70°; voxel size = 2 mm isotropic; imaging matrix = 106 × 106; FOV = 212 mm. 72 slices were oriented in the transversal plane covering the entire brain. A total of 216, 172 and 346 volumes were collected per participant for each run of the body, face and finger tasks respectively. Field-maps were acquired for field unwarping. A T1-weighted sequence (MPRAGE, TR = 2530 ms; TE = 3.34 ms; flip angle = 7°; voxel size = 1 mm isotropic) was used to obtain anatomical images.

QUANTIFICATION AND STATISTICAL ANALYSIS

MRI analysis was implemented using tools from FSL (v. 6.00, Smith et al., 2004; Jenkinson et al., 2012), Connectome Workbench software (v. 1.4.2, humanconnectome.org) in combination with bash and Matlab scripts (v. R2016a, mathworks.com), both developed in-house and as part of the RSA Toolbox (Nili et al., 2014). Cortical surface reconstructions were produced using FreeSurfer (v. 7.1.1; Dale et al. 1999; Fischl et al. 2001, freesurfer.net).

fMRI pre-processing

Functional data was pre-processed in FSL-FEAT (v. 6.00) and included the following steps: motion correction using MCFLIRT (Jenkinson et al., 2002); brain extraction using BET (Smith, 2002); high-pass temporal filtering with a cut-off of 150s, 119s and 150s for the body, face and finger tasks respectively and 280s for the functional localiser; and finally spatial smoothing using a Gaussian kernel with a full width at half maximum of 3 mm for the three tasks, and 5 mm for the functional localiser. Field maps were used for distortion correction of the functional data from the body and face tasks and the functional localiser collected for these participants. For each participant, a midspace between the different functional runs of each task was calculated, i.e., the average space in which the images are minimally reorientated. Each functional run was then aligned to the midspace and registered to each individual structural T1 scan using FMRIB's Linear Image Registration Tool (FLIRT), optimised using Boundary-Based Registration (Greve and Fischl, 2009). Where specified, functional and structural data were transformed to MNI152 space using FMRIB's Nonlinear Registration Tool (FNIRT).

fMRI low-level analysis

Voxel-wise General Linear Model (GLM) was applied to the data using FEAT to obtain statistical parametric maps for each movement. For each task, the design comprised a regressor of interest for each movement convolved with a double-gamma hemodynamic response function (Friston et al., 1998) and its temporal derivative. The six motion parameters were included as regressors of no

CellPress
OPEN ACCESS

interest. Large head movements between volumes (>0.9 mm for body and face tasks, > 1 mm for finger task) were defined as motion outliers and regressed out, by adding additional regressors of no interest to the GLM [body task: mean proportion of volumes excluded = 0.45 ± 0.76 (SD) %; face task: mean proportion of volumes excluded = 0.36 ± 0.67 (SD) %; finger task: mean proportion of volumes excluded = 0.32 ± 0.77 (SD) %].

For each task, a contrast relative to rest was set up for each movement, resulting in 8 contrasts for the body task (i.e., lip, dominant hand, non-dominant arm, right/left foot x Squeeze or Push, each vs rest), in 6 contrasts for the face task (i.e., forehead, nose, lips, tongue; and left/right thumb, not used here), and in 12 contrasts for the finger task (i.e., each digit of each hand; and feet and lips, not used here). The estimates from the total number of functional runs for each task (3 for face task, 4 for body and finger tasks) were then averaged voxel-wise at the individual level using fixed effects model. For the face task, each estimates' average was masked prior to cluster formation with a sensorimotor mask, defined as the precentral and postcentral gyrus from the Harvard Cortical Atlas. The sensorimotor mask was registered to the individuals structural scan using an inversion of the nonlinear registration by FNIRT.

For the functional localiser, each condition (i.e., right/left hand, right/left toes, lips) was contrasted against all other conditions to identify the most selective voxels. The activity patterns associated with these five contrasts were then registered to the structural space of each individual and to the functional space of each task using FLIRT to define regions of interest.

Definition of regions of interest (ROIs)

Since we were interested in investigating the information content of highly selective regions across the S1 homunculus, we used the functional localiser to select highly selective voxels to toe, hand and lip movements within anatomical S1 masks. The functional ROI was restricted by anatomical criteria, as detailed below. Although M1 is expected to be largely activated during each movement, M1 topography tends to be less well-defined and thus information content more widespread (Schieber, 2001; Graziano and Aflalo, 2007). We therefore primarily focus on the more topographically selective S1, though we wish to note that marginal contribution from M1 may have affected our S1 activity profiles due to their spatial proximity, the probabilistic nature of our anatomical masks and spatial smoothing of the data.

First, S1 was defined on a template surface using probabilistic cytoarchitectonic maps, by selecting only nodes that belonged to the grey matter of Brodmann areas (BAs) 3a, 3b, 1 and 2 with maximal probability (Wiestler and Diedrichsen, 2013). This S1 anatomical mask was then split into three anatomical sub-regions. A node approximately 1 cm below and above the hand knob was defined as an anatomical hand sub-region. Note that this criterion defined a more conservative hand region than was done in previous work (Wiestler and Diedrichsen, 2013; Wesselink et al., 2019; Kieliba et al., 2021). A gap of 1 cm was then defined above and below this anatomical hand sub-region, and the remaining medial and lateral parts of S1 were used as the other two anatomical sub-regions.

Structural T1-weighted images were used to reconstruct the pial and white-grey matter surfaces using Freesurfer. Surface coregistration across hemispheres was done using spherical alignment. The three anatomical S1 sub-regions were then projected into the individual brains via the reconstructed individual anatomical surfaces. To exclude any possible contribution from neighbouring more integrative regions that contain information from multiple body parts, we further trimmed in participant's structural space: i) the medial sub-region by removing any overlap with BA5L and BA5M, and ii) the lateral sub-region by removing any overlap with S2. BA5L, BA5M and S2 were defined in MNI152 space using the Juelich Histological Atlas thresholded at 25% maximum probability (Wiech et al., 2014). BA5L, BA5M and S2 were then registered to participants' structural space using an inversion of the nonlinear registration carried about by FNIRT and used to trim our anatomical sub-regions.

These trimmed anatomical sub-regions were then registered to functional space of each task using FLIRT, excluding voxels partially overlapping with the central sulcus (thresholding at 0.5 for body and face tasks, and 0.2 for the finger task) to minimise M1 contribution, and used to mask the functional localiser contrasts. The medial S1 sub-region was used to mask the toe contrasts, the central hand sub-region to mask the hand contrasts and the lateral S1 sub-region to mask the lip contrast. Within each of these anatomical sub-regions, we then selected the 50 most activated voxels for the corresponding contrasts (all contrasts vs all other body parts, see section 'fMRI low-level analysis'). This provided us with the most selective Leg, Hand and Face ROIs for each individual, while ensuring the same ROI size across participants and regions.

Univariate analysis

The z statistic time series from the 50 voxels of each ROI obtained for each movement were extracted and averaged. These averaged values were used to assess the selectivity of our ROIs. Univariate information content was defined as the absolute difference between the averaged univariate activity evoked by two movements in a given ROI. For the body task, the two absolute differences obtained between pairs of body parts when performing the same action (i.e., squeeze or push) were averaged to define an overall difference between body parts. For the face and finger tasks, since face movements evoked bilateral activity and finger movements were performed with each hand, and since no major differences were observed across hemispheres [three-way ANOVA Face task: all $F \leq 1.30$, all $p \geq 0.267$, except for a triple interaction Hemi*ROIs*Face subparts ($F_{(3.12,65.49)} = 3.06$, $p = 0.033$) revealing a significant difference between hemispheres for the lips ($z_{(21)} = 45.00$, $p = 0.007$) and for the nose in the Leg ROI only ($t_{(21)} = -2.78$, $p = 0.011$); three-way ANOVA Finger task: all $F \leq 1.20$, all $p \geq 0.293$], absolute differences from the two hemispheres were averaged within

participants. To further reduce the number of comparisons while still assessing the topographical content, absolute difference from different pairs of subparts (i.e., face parts or fingers) were grouped according to the subpart's cortical neighborhood (i.e., adjacent vs non-adjacent).

Multivariate representational similarity analysis

Representational Similarity Analysis (RSA; Nili et al., 2014) was used to assess the multivariate relationship between the contralateral activity patterns generated by each movement. The dissimilarity between activity patterns within each S1 ROI (i.e., Leg, Hand and Face) was computed at the individual level for each pair of movements using cross-validated squared Mahalanobis distance (Walther et al., 2016). Multidimensional noise normalisation was used to increase reliability of distance estimates (noisier voxels are down-weighted), based on the voxel's covariance matrix calculated from the GLM residuals. Due to cross-validation, the expected value of the distance is zero (or negative) if two patterns are not statistically different from each other, and significantly greater than zero if the two representational patterns are different (Diedrichsen et al., 2016). Larger distances for movement pairs therefore suggest greater information content. The resulting representational pairwise distances (i.e., 8 for the body task, 6 for the face task and 10 for the finger task) were extracted. For the body task, the dissimilarities obtained between pairs of body parts when performing similar actions (e.g., dissimilarity between lip squeeze and feet squeeze) were averaged across actions (e.g., previous example averaged with dissimilarity between lip push and feet push) to define overall dissimilarity between body parts. For the face and finger tasks, since face movements evoked bilateral activity and finger movements were performed with each hand, and since no major significant differences were observed between hemispheres (see above), dissimilarities from the two hemispheres were averaged within individual participants. To further reduce the number of comparisons while still assessing the topographical content, dissimilarities from different pairs of subparts (i.e., face parts or fingers) were grouped according to the subpart's cortical neighborhood (i.e., adjacent vs non-adjacent). Multidimensional scaling (MDS) was used to project the higher-dimensional representational dissimilarity matrices into lower-dimensional space, whilst preserving pairwise dissimilarities, for visualisation purposes only. To illustrate what activity the RSA analysis is relying upon, we computed the unthresholded S1 univariate maps of participants showing dissimilarities close to the group median for the different contents of interest (e.g., median lips-hand dissimilarity in the Leg ROI for the body task, Figure S5A). Analysis was conducted on an adapted version of the RSA Toolbox in MATLAB (Nili et al., 2014), customised for FSL (Wesselink and Maimon-Mor, 2018).

BA3b analysis

The same analyses as the ones performed in the S1 ROIs described above were performed throughout BA3b's strip. BA3b was defined on the same template surface as S1 using probabilistic cytoarchitectonic maps, by selecting the nodes showing at least 50% maximum probability for the grey matter of BA3b (Wiestler and Diedrichsen, 2013). Due to the use of surface-based ROIs where BA3b does not directly neighbour M1 (BA4p), this analysis is unlikely to be contaminated by M1 contributions. BA3b's strip was then segmented into 30 bands, each 2.09 mm high in the medio-lateral direction (see Figure 5A). Similar to the S1 ROIs, these bands were then projected into the individual brains via the reconstructed individual anatomical surfaces and registered to participants' functional space of each task using FLIRT. On average, each band contained 46.12 voxels ±16.22 (SD). Univariate and multivariate analyses were then repeated in each of these bands. Note that the most medial band contained very few and discontinuous voxels that prevented from getting reliable RSA dissimilarities, and was thus excluded from further analysis.

Statistical analysis

All statistical analyses were carried out using JASP (v. 0.14). Two-tailed one-sample t-tests versus zero were used to assess significant activity levels in each ROIs. Alpha levels were Bonferroni corrected for the number of tests performed across conditions within each ROI (i.e., alpha = 0.017 corrected for three comparisons for the body task, alpha = 0.013 corrected for four comparisons for the face task and alpha = 0.010 corrected for five comparisons for the finger task). Since negative dissimilarity measures represent noise levels, one-tailed one-sample t-tests versus zero were used to test the significance of representational dissimilarities as well as absolute differences in activity levels in each ROIs. Here again, alpha levels were Bonferroni corrected for the number of tests performed within each ROI (i.e., corrected for three comparisons for the body and action dissimilarities, and for two comparisons for the adjacent and non-adjacent dissimilarities for the face and finger tasks). Paired t-tests were used to compare adjacent and non-adjacent conditions for the face and finger tasks. In each case, a trend was defined when p values were inferior to twice the corrected alpha level. Data normality was assessed using Shapiro-Wilk test. Effect sizes were computed using Cohen's *d* (Cohen, 1988), and based on benchmarks suggested by Cohen, a large effect size was defined as greater than 0.8. Wilcoxon signed-rank t-tests were used when data violated normality assumptions. Two three-way rmANOVAs with the factors Hemisphere, ROI and Subpart were applied to univariate activity levels from the face and finger tasks to assess Hemisphere effect or interaction. Since no major differences were observed across hemispheres, univariate and multivariate data from each hemisphere were averaged. Greenhouse-Geisser correction was applied when data did not follow sphericity assumption. All group data are expressed as means ± SEM, except mentioned otherwise.

CellPress
OPEN ACCESS

Group-level ROI visualisations

S1 ROIs of each participant were projected to MNI152 space using the nonlinear registration carried about by FNIRT. Participant information regarding hand dominance were used to sagittal-flip data, such that the ROIs contralateral to the dominant hand were always represented in the left hemisphere. ROIs of all participants were then concatenated into a single volume to produce a consistency map (i.e., how many participants have their ROIs overlapping in the MNI space). Resulting consistency maps were then projected to a group cortical surface (Glasser et al., 2016) using Connectome Workbench (v1.4.2) (see Figures 1 and S2 for body task; see Figure S3B for face and finger tasks).

Cell Reports, Volume *38*

Supplemental information

Beyond body maps: Information content of specific body parts is distributed across the somatosensory homunculus

Dollyane Muret, Victoria Root, Paulina Kieliba, Danielle Clode, and Tamar R. Makin

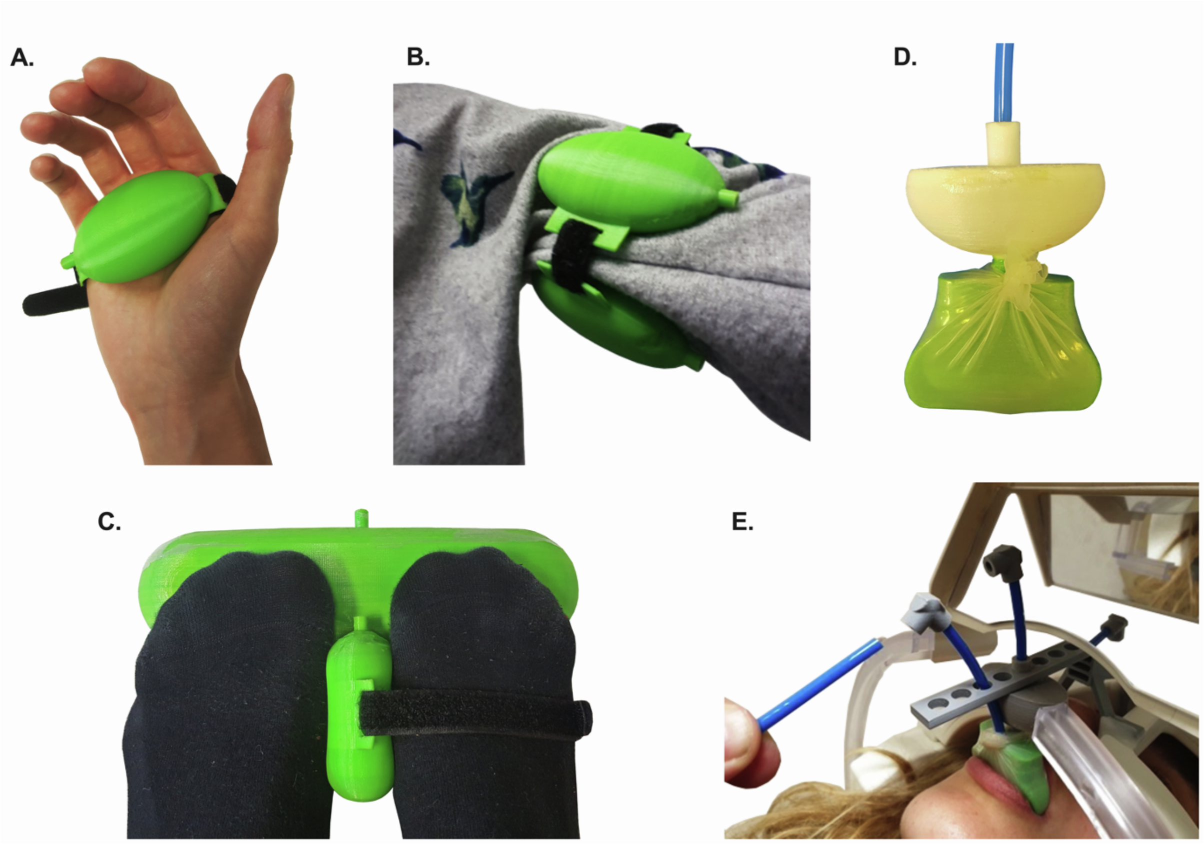

Figure S1. Objects used in the body task. Related to Figure 1, 4 and 5, and to STAR Methods. Silicon 3D printed objects (NinjaFlex© 3D printing filament) were placed and secured with straps in the participants dominant hand (A), around the non-dominant arm (B), between the feet (secured at the level of their right metatarsophalangeal joints), and behind their toes (strapped to a footrest secured on the bed behind their feet) (C). Participants were instructed to either squeeze (i.e., closing the fingers for the hand, against the torso for the arm and between the feet) or push (i.e., against the bed for hand and arm, and against the footrest for the feet) the object with each body part. Three participants also had an object placed in between their lips (D), and secured on a fully adjustable frame, fixed on the coil (E), object that could be either squeezed or pushed. The other participants were asked to either purse their lips (equivalent to squeezing) or blow kisses (equivalent to pushing). A plastic tube was connecting each of the objects to a transducer system in the control room, allowing us to monitor and quantify the amount of pressure exerted.

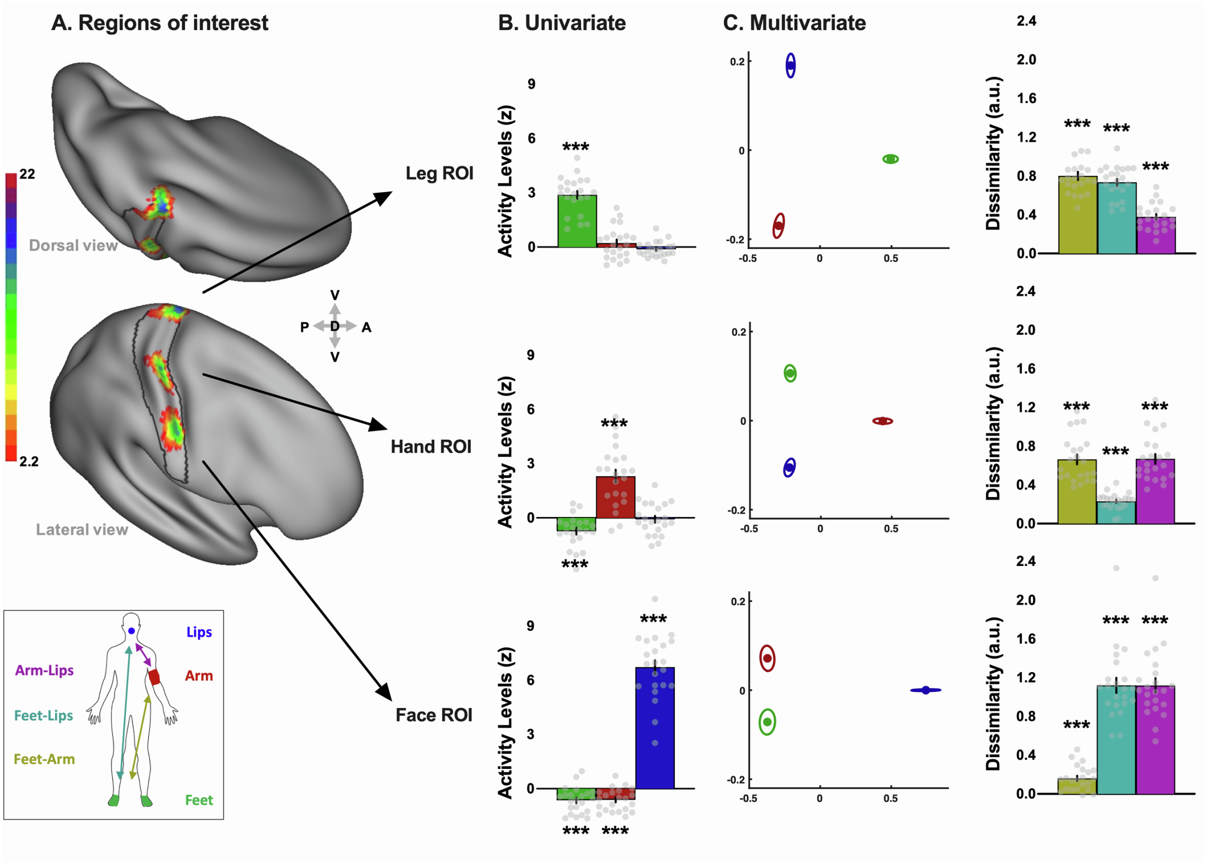

Figure S2. Regions of interest, selectivity, and multivariate information content related to specific body parts across S1 Homunculus in the non-dominant hemisphere. Related to Figure 1. All figure annotations are as denoted in Figure 1. Asterisks indicate a significant difference relative to zero; ***$p < 0.001$. In agreement with what was found in the dominant hemisphere (Fig. 1), ROIs in the non-dominant hemisphere were highly selective to their primary body parts, showing activity levels significantly above zero for this body part only (primary body parts: all $t \geq 6.50$, all $p < 0.001$, all $d \geq 1.39$ CI [0.88 1.87]; non-primary body parts: all $t \leq 1.20$, all $d \leq 0.25$ CI [-0.10 0.61]). And here again, the dissimilarity between activity patterns evoked by each movement were significantly greater than zero not only for pairs of body parts involving the primary body part of each ROI (all $t \geq 13.01$, all $p < 0.001$, all $d \geq 2.77$ 95% CI [1.97 ∞]), but also for pairs of body parts non-primary to the ROI (all $t \geq 5.84$, all $p < 0.001$, all $d \geq 1.25$ 95% CI [0.76 ∞]).

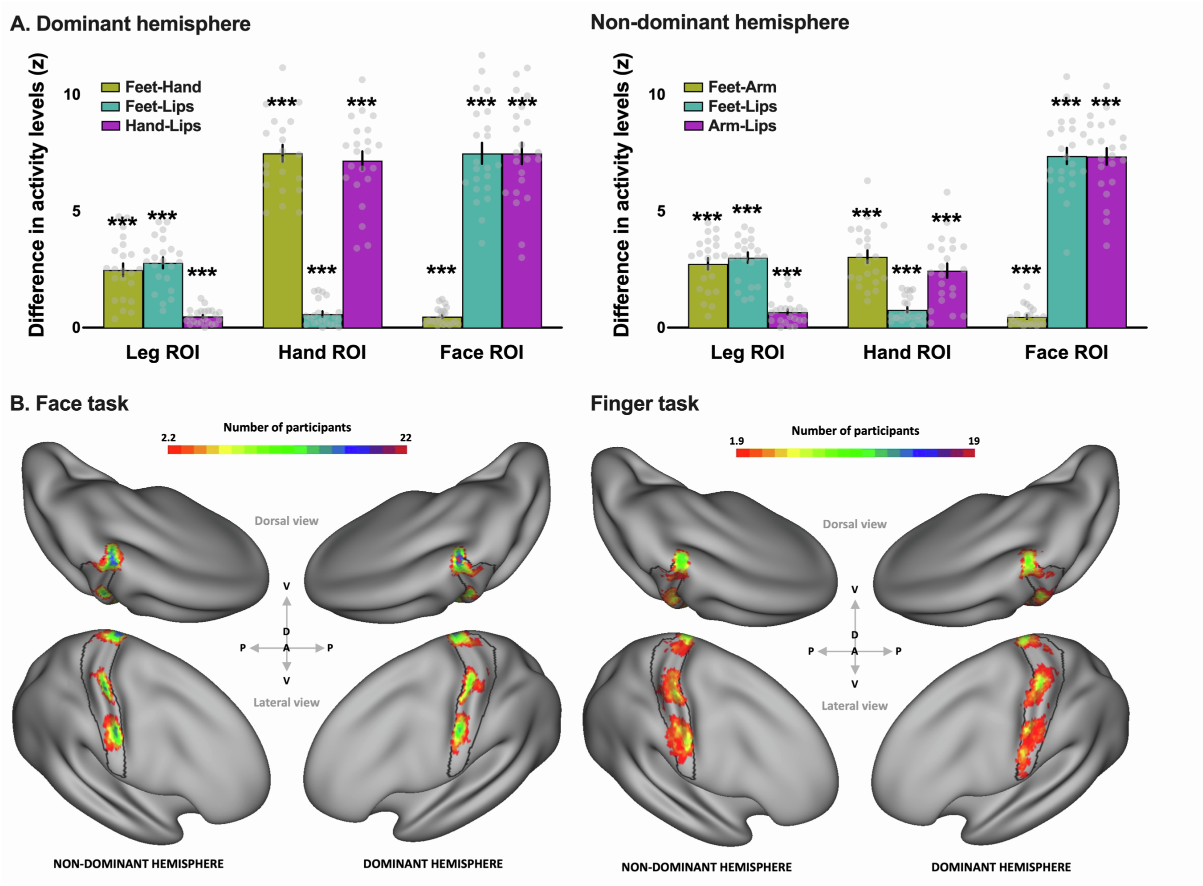

Figure S3. Univariate information content related to specific body parts across the S1 Homunculus and regions of interest for the face and finger tasks. Related to Figure 1A&C. A) Absolute differences between the univariate activity levels observed for each pair of body parts (yellow: feet-hand/arm, cyan: feet-lips, magenta: hand/arm-lips) across S1 ROIs in the hemisphere contralateral to the dominant hand (left) or non-dominant arm (right). In each hemisphere absolute differences were significantly greater than zero not only for pairs of body parts involving the primary body part of each ROI (all $t_{(21)} \geq 7.96$, all $p < 0.001$, all $d \geq 1.70$ 95% CI [1.13 ∞]), but also for pairs of body parts that were non-primary to the ROI (all $t_{(21)} \geq 5.18$, all $p < 0.001$, all $d \geq 1.10$ 95% CI [0.65 ∞]). Grey dots represent individual participants. ***$p < 0.001$. **B)** Consistency maps across participants of regions of interest within the primary somatosensory cortex (S1) used for the face (left) and finger (right) tasks respectively. Same legend as Fig. 1A and Fig. S2A.

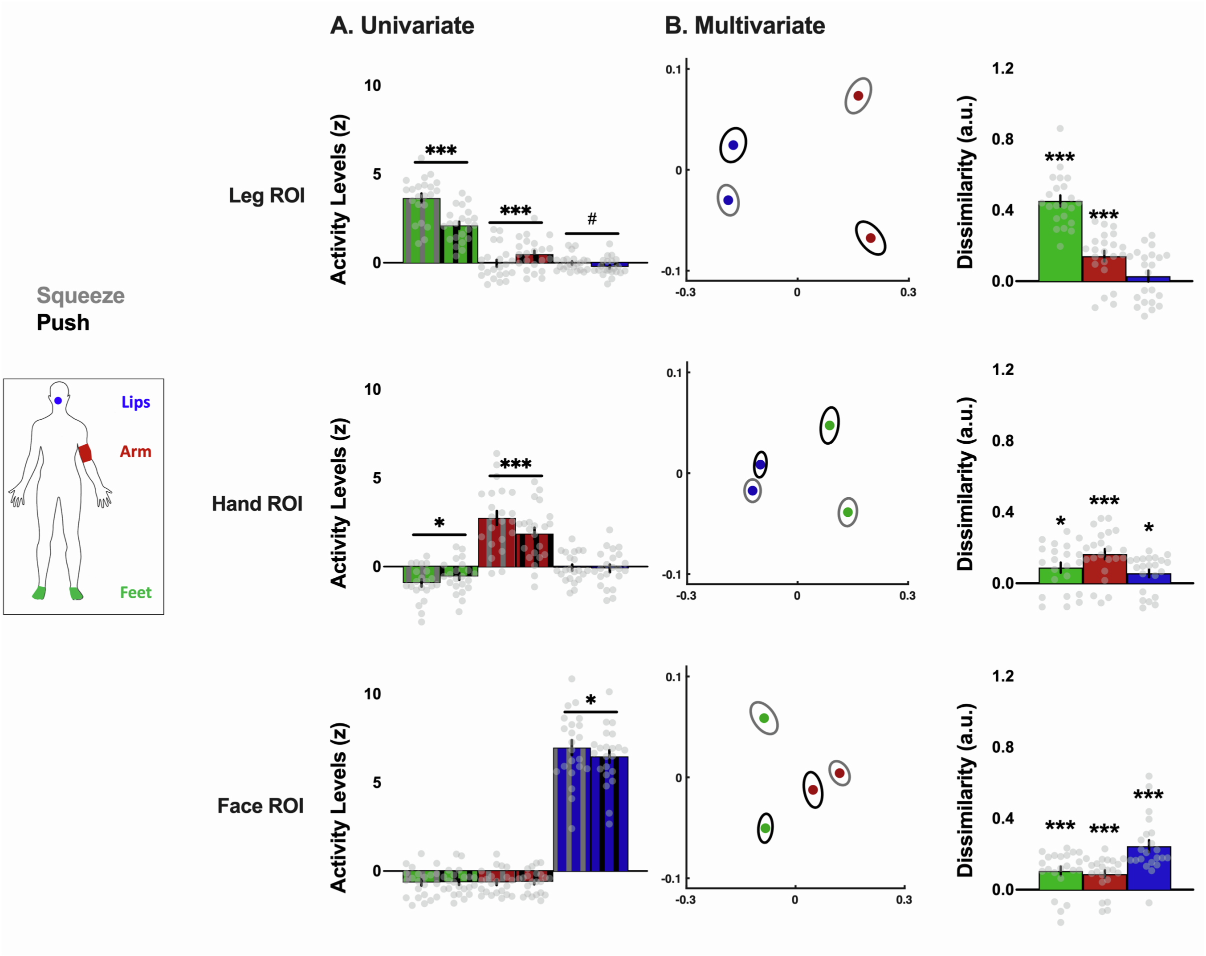

Figure S4. Univariate and multivariate content related to different actions performed with a given body part across S1 Homunculus in the non-dominant hemisphere. Related to Figure 4. All annotations are as denoted for Figure 4. *$p < 0.017$; $^{\#}p < 0.033$; ***$p < 0.001$. In agreement with what was found in the dominant hemisphere (Fig. 4), activity levels evoked by different actions were significantly different in the primary ROIs of each body part (all $p \leq 0.013$). Significant differences were also observed for arm movements in the Leg ROI ($t_{(21)} = -6.04$, $p < 0.001$) and for feet movements in the Hand ROI ($t_{(21)} = -2.67$, $p = 0.014$), and a trend was found for lip movements in the Leg ROI ($t_{(21)} = 2.44$, $p = 0.024$; other conditions: $-0.27 <$ all $t_{(21)} < 0.36$, all $p \geq 0.722$). Similar to the results observed in the dominant hemisphere, multivariate representational dissimilarities were significantly greater than zero not only in primary ROIs (all p values < 0.001, all $d \geq 0.99$ CI [0.98 ∞]), but also in non-primary ROIs for arm and feet movements (all $p \leq 0.003$, all $d \geq 0.66$ CI [0.26 ∞]), and for lip movements in the Hand ROI ($z_{(21)} = 196$, $p = 0.011$, $d = 0.55$ CI [0.21 ∞]).

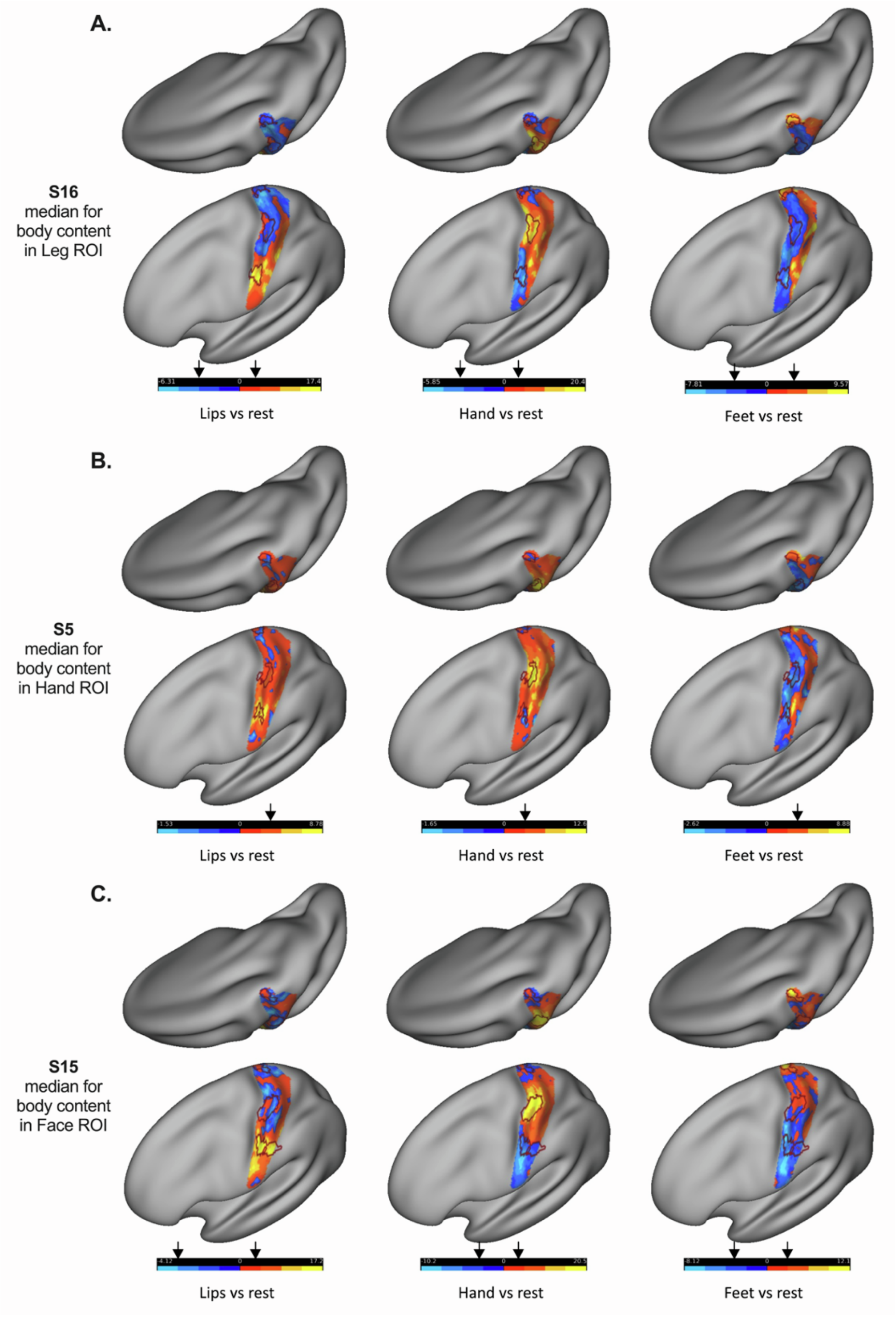

Figure S5. Example individual univariate activity maps obtained for different body parts across S1 in the dominant hemisphere. Related to Figure 1. Participants showing the closest dissimilarity to the group median for the body content in **A)** the Leg ROI, **B)** the Hand ROI and **C)** the Face ROI. Body content is defined as the dissimilarity between non-primary body parts. The colour scale codes for the z statistic (contrast vs rest), with positive bold responses represented in red-yellow and negative bold responses in blue. Standard activity threshold (z = 3.1) is indicated with the black arrows on the activity scales. Individual's ROIs are highlighted with red contours. The data is not thresholded and masked with S1 anatomical ROI. We note that the univariate activity profile was providing inconsistent results (across participants and paradigms), and thus these unthresholded maps of single participants should be interpreted with much caution.
